# The Characterization of a New AG-II-like Glycoprotein from *Cynanchum thesioides* (Freyn) K. Schum and Its Immunostimulatory Activity Through Activation of TLR4/9-Mediated MAPK/NF-κB Signaling Pathways

**DOI:** 10.3390/cimb48080804

**Published:** 2026-08-08

**Authors:** Mu Dan, Peng Zhao, Lu Ga, Wenming Bai, Pengwei Zhao, Han Ge, Ruirui Wang, Surina Bo, Munkhtsetseg Baatar

**Affiliations:** 1 Department of Chemical and Biological Engineering, School of Engineering and Technology, National University of Mongolia, Ulaanbaatar 14201, Mongolia; mudan97@163.com; 2 College of Pharmacy, Inner Mongolia Medical University, Jinshan Development Zone, Hohhot 010110, China; 15232111906@163.com (P.Z.); 13404832082@163.com (L.G.); 20230007@immu.edu.cn (W.B.); 3 College of Basic Medicine, Inner Mongolia Medical University, Jinshan Development Zone, Hohhot 010110, China; pengwzhao@126.com; 4 Shanghai Innovation Center of TCM Health Service, Shanghai University of Traditional Chinese Medicine, Shanghai 201203, China; gehanhange@163.com (H.G.); wangruirui@shutcm.edu.cn (R.W.); 5 State Key Laboratory of Integration and Innovation of Classic Formula and Modern Chinese Medicine, Shanghai University of Traditional Chinese Medicine, Shanghai 201203, China

**Keywords:** *Cynanchum thesioides* (Freyn) K. Schum, glycoprotein, structural characterization, immunostimulatory activity, signaling pathways

## Abstract

The structural and immunomodulatory properties of arabinogalactan proteins (AGPs) from edible medicinal plants remain largely unexplored. Here, A homogenous AG-II-like arabinogalactan protein (CTSP-W2, 9862 Da) was isolated from *Cynanchum thesioides* via hot-water extraction, ethanol precipitation, and column chromatography. Its structure was thoroughly characterized by high-performance gel permeation chromatography (HPGPC), Fourier-transform infrared spectroscopy (FT-IR), nuclear magnetic resonance (NMR), Congo-red, scanning electron microscopy (SEM), sodium dodecyl sulfate-polyacrylamide gel electrophoresis (SDS-PAGE), methylation analysis. The mechanism of immune activity was examined using specific inhibitors, Western blotting, and molecular docking. It comprises galactose, arabinose, glucose, galacturonic acid, xylose, and 18 amino acids (asparagine-rich), with a backbone of →3,6)-Galp-(1→ and →6)-Galp-(1→. CTSP-W2 significantly enhanced macrophage proliferation, phagocytosis, and secretion of Nitric oxide (NO), Tumor necrosis factor-alpha (TNF-α), and Interleukin-6 (IL-6). Inhibitor assays showed that Toll-like receptor 4 (TLR4, TAK-242) and Toll-like receptor 9 (TLR9, E6446) antagonists markedly reduced CTSP-W2-induced TNF-α, IL-6, and NO in a dose-dependent manner, whereas Toll-like receptor 2 (TLR2) inhibition (C29) unexpectedly upregulated these mediators. Western blot revealed that CTSP-W2 upregulated TLR4 and TLR9 protein expression and increased phosphorylation of Inhibitor of nuclear factor kappa-B alpha (IκBα), nuclear factor kappa B (NF-κB p65), and p38, indicating activation of the TLR4/9–NF-κB–p38 mitogen-activated protein kinase (MAPK) signaling axis. Furthermore, Molecular docking analysis further indicated that CTSP-W2 forms extremely strong hydrogen-bonding and hydrophobic interactions with TLR4 through its galactose chains. This study elucidates the immunoregulatory mechanism of CTSP-W2 and establishes a molecular basis for arabinogalactan proteins as potential natural immunomodulators.

## 1. Introduction

*Cynanchum thesioides* (Freyn) K. Schum (*C. thesioides*), a member of the genus *Cynanchum* in the family Asclepiadaceae, is widely distributed in sandy areas of northeastern China, northern China, and Inner Mongolia [1]. As a medicinal plant, its fruit is edible, and the whole herb is used therapeutically. *C. thesioides* is rich in polysaccharides, unsaturated fatty acids, proteins, dietary fiber, vitamins, and minerals, which contribute to the regulation of physiological functions and the promotion of overall health. Therefore, it is considered a high-quality raw material for the development of functional and specialty health foods [1]. In traditional Chinese and Mongolian medicine, *C. thesioides* is traditionally used to replenish qi (vital energy), nourish qi and yin (maintain the balance of vital energy and body fluids), and treat postpartum weakness and insufficient lactation [2]. Previous studies have also shown that the water extracts of *C. thesioides* exhibits inhibitory activity against various pathogenic bacteria and can effectively repair intestinal mucosal damage [3]. Despite its broad clinical applications, research on the active constituents of *C. thesioides* remains limited, which constrains its further development and utilization in medicinal and health-related products.

Glycoproteins are biomolecular conjugates formed through the covalent linkage of glycan chains to peptides or proteins. These complex biomolecules display diverse structural features and exert important biological activities, including immunomodulation, antitumor activity, antioxidant effects, and anti-inflammatory properties [4]. Both in vitro and in vivo immunoreactivity studies have demonstrated that glycoproteins can enhance and modulate organismal immune function [5]. For example, glycopeptides extracted from *Trametes versicolor* are clinically employed as immunopotentiators [6]. Glycoproteins isolated from the traditional medicinal herb *Cirsii herba* [7], mountain-cultivated ginseng [5], and natto (fermented soybeans) [8], have been shown to stimulate cytokine secretion by antigen-presenting cells and to enhance immunomodulatory activity through the MAPK and NF-κB signaling pathways, mediated by interactions with Toll-like receptors (TLRs). TLRs are evolutionarily conserved molecules that recognize pathogens and initiate immune responses. They play a crucial role not only in activating innate immune reactions but also in bridging innate and adaptive immune responses [9].

Arabinogalactans from *Ixeris chinensis* (58.1 kDa), jasmine processing waste (JSP-1a, 87.5 kDa), *Ficus pumila* fruit hulls (FPW, 156 kDa), and *B. carterii* (295.5 kDa) consistently activate RAW264.7 macrophages via TLR4/TLR2-mediated NF-κB and MAPK pathways, promoting phagocytosis and the release of NO, TNF-α, IL-6, and interleukin-1 beta (IL-1β). Structural elucidation reveals that these polysaccharides share conserved type II arabinogalactan backbones composed of β-(1→3)- and β-(1→6)-linked Galp residues with O-6 branching, along with non-reducing terminal Araf and Galp units [10,11,12,13]. Nevertheless, the precise structure–activity relationship (SAR) remains inadequately defined. Emerging evidence suggests that molecular weight—with lower fragments around 10 kDa often-displaying higher activity—and the degree of branching, especially the proportion of →3,6)-Galp branch points, critically influence receptor cross-linking efficiency and downstream signaling amplification. However, systematic quantitative studies correlating specific structural parameters (e.g., branch density, side-chain length, terminal epitopes) with immunopotency are still lacking. Furthermore, most reported arabinogalactans originate from limited plant families, and the utilization of underutilized agricultural by-products as sustainable polysaccharide sources remains largely unexplored. Hence, in this study, we isolate and structurally characterize a novel arabinogalactan from *C. thesioides*, determine its glycosidic linkages via comprehensive methylation and NMR analyses, evaluate its immunomodulatory effects on RAW264.7 macrophages, and propose a preliminary SAR framework.

Polysaccharides from several *Cynanchum* species have demonstrated potent immunomodulatory activities. *C. wilfordii* polysaccharides enhance immune function in macrophages and immunosuppressed mice while alleviating colitis [14]. *C. auriculatum* polysaccharides promote phagocytosis and cytokine secretion via NF-κB activation [15]. *C. atratum* arabinogalactan (CAPW-1, 64 kDa) stimulates macrophage proliferation and NO/TNF-α/IL-6 production through the TLR4/NF-κB/vascular endothelial growth factor C (VEGF-C) pathway and induces tolerogenic dendritic cells [16]. Despite these findings, the structural features and immunomodulatory mechanisms of arabinogalactan proteins (AGPs) from *C. thesioides* remain completely unexplored. As a xerophytic medicinal herb and food source native, *C. thesioides* represents a promising yet overlooked resource for natural immunomodulators in post-vaccination and geriatric populations, warranting systematic investigation of its AGP structure and TLR-mediated activity.

Mammalian Toll-like receptors (TLRs) serve as primary sentinels for recognizing pathogen- and damage-associated molecular patterns. Among them, TLR4 and Toll-like receptor 9 (TLR9) activate distinct intracellular adapter pathways-TLR4 recruits both Myeloid differentiation primary response protein 88 (MyD88) and Toll/IL-1 receptor domain-containing adaptor-inducing interferon-β (TRIF) to induce NF-κB- and interferon regulatory factor 3 (IRF3)-mediated pro-inflammatory cytokines (TNF-α, IL-6) and type I interferons, whereas TLR9 primarily signals through MyD88, yielding a different cytokine profile. This pathway divergence is pharmacologically significant for drug development [17,18,19]. Notably, TLR2 typically recognizes bacterial lipoproteins rather than polysaccharides.; thus, its involvement in plant AGP recognition is less intuitive.

Herein, we aimed to isolate a homogenous glycoprotein (CTSP-W2) from *C. thesioides*, systematically characterize its structure and morphology, and evaluate its immunomodulatory effects and mechanism on macrophages in vitro. These results will help clarify the immunoregulatory activity of CTSP-W2 and provide a theoretical foundation for the development and application of *C. thesioides* in functional foods and pharmaceutical products.

## 2. Materials and Methods

### 2.1. Materials and Chemicals

*C. thesioides* was harvested in the summer of 2024 from Chifeng city, Inner Mongolia, China. DEAE-cellulose 52, monosaccharide standards, and a methylation kit were purchased from Yangzhou Borui Sugar Biotechnology Co., Ltd. (Yangzhou, China). Pullulan standards were procured from Showa Denko (Minato-ku, Tokyo, Japan). Coomassie Brilliant Blue R-250, periodic acid–Schiff reagent, and sodium dodecyl sulfate (SDS) were acquired from Bio-Rad (Hercules, CA, USA), and Protein molecular weight markers for SDS-PAGE were obtained from Thermo Fisher Scientific (Waltham, MA, USA). Roswell Park Memorial Institute 1640 (RPMI-1640) medium, fetal bovine serum (FBS), penicillin–streptomycin solution, and trypsin were acquired from Gibco (Grand Island, NY, USA). A Cell Counting Kit-8 (CCK-8) was purchased from Dojindo Molecular Technologies (Kumamoto, Japan). A bicinchoninic acid (BCA) protein assay kit and mouse TNF-α, IL-1β, and IL-6 enzyme-linked immunosorbent assay (ELISA) kits were supplied by Dakewe Biotech (Beijing, China). A mouse NO assay kit was purchased from Xinqidi Biological Technology (Wuhan, China). The murine macrophage cell line RAW 264.7 (Cat. No. SCSP-5036) was obtained from the Cell Bank of the Chinese Academy of Sciences (Shanghai, China). Primary antibodies against TLR4, phosphorylated nuclear factor kappa B p65 (p-NF-κB p65), total NF-κB p65, phosphorylated p38 mitogen-activated protein kinase (p-p38 MAPK), total p38 MAPK, phosphorylated inhibitor of nuclear factor kappa B (p-IκB), and total IκB, as well as radioimmunoprecipitation assay (RIPA) lysis buffer, were purchased from Proteintech (Wuhan, China). Antibodies against TLR9 and β-actin were obtained from Bioss (Woburn, MA, USA). Protease inhibitor and phosphatase inhibitor cocktails were purchased from Yamay Biomedical Technology (Shanghai, China). Bovine serum albumin (BSA) and lipopolysaccharide (LPS) derived from Escherichia coli O55:B5 were obtained from Solarbio (Beijing, China). Protease inhibitor and phosphatase inhibitor cocktails were purchased from Yamay Biomedical Technology (Shanghai, China). BCA and 5% bovine serum albumin (BSA) were obtained from Solarbio (Beijing, China).

### 2.2. Isolation and Purification of CTSP-W2

The whole plant of *Cynanchum thesioides* was naturally air-dried in a shaded and well-ventilated environment for approximately 7 days, pulverized, and passed through a 60-mesh sieve. One hundred grams of the powder was soaked in 500 mL of distilled water overnight at room temperature and extracted under reflux at 100 °C for 1 h using a temperature-controlled electric heating mantle (ZDHW, Shandong Sangze Instrument Co., Ltd., Jining, China). The extraction was repeated three times, and the filtrates were combined. The extract was concentrated under reduced pressure, precipitated with 80% ethanol overnight, centrifuged at 4000 rpm, and dried at 65 °C to obtain the glycoprotein-enriched fraction (CTSP), and the extraction yield was 10.28 ± 0.45%. The Sevag method was applied to remove free proteins from the crude glycoprotein extract. Following deproteinization, 68 g of the crude glycoprotein sample (0.17 g/mL) was subjected to further purification using a DEAE-cellulose 52 column (Solarbio, Beijing, China; 4 cm internal diameter × 60 cm height).

Prior to sample loading, the column was pre-equilibrated with 0.5 mM hydrochloric acid for 1 h at a flow rate of 5 mL/min. The column was subsequently washed with 4–5 column volumes of distilled water until the effluent reached a neutral pH, followed by equilibration with distilled water for an additional 2 h at the same flow rate, which was controlled using a peristaltic pump system consisting of a pump head (YZ1515X, Baoding Longer Precision Pump Co., Ltd., Baoding, China) and a pump driver (CM3000, Baoding Chuangrui Pump Industry Co., Ltd., Baoding, China). The prepared sample was then loaded onto the equilibrated column for purification. Elution was then performed sequentially with three column volumes of distilled water and NaCl solutions of increasing concentrations (0.2, 0.5, and 1.0 M) at a flow rate of 1.5 mL/min. Neutral polysaccharides were eluted with distilled water, followed by stepwise elution with 0.2, 0.5, and 1.0 M NaCl solutions to obtain acidic polysaccharide fractions. The elution process was monitored using the phenol-sulfuric acid method at 490 nm. The collected eluates were dialyzed using a dialysis membrane with a molecular weight cutoff of 3500 Da under running tap water for 48 h, followed by dialysis against distilled water for 12 h. The dialyzed samples were subsequently concentrated and freeze-dried to obtain fractions designated CTSP-W, CTSP-0.2M, CTSP-0.5M, and CTSP-1.0M. The yield of each fraction was calculated. Subsequently, 6.0 g of CTSP-W (0.25 g/mL) was further separated using a HPGPC Auto Purifier System-2 (BRT-GS, Bo Rui Saccharide Biotech, Yangzhou, China) equipped with a Sephadex G-100 column (110 cm × 2.4 cm), a refractive index detector (RI-10A, Shimazu, Japan), and an automatic fraction collector. Elution was conducted with distilled water at a flow rate of 1.5 mL/min for 150 min. Elution profiles were detected online, and fractions corresponding to symmetrical peaks were collected. These fractions were dialyzed, concentrated, and freeze-dried to obtain homogenous polysaccharides, which were confirmed through high-performance gel permeation chromatography (HPGPC).

### 2.3. SDS Gel Electrophoresis

The purity and molecular mass of the CTSP-W2 fraction were determined via SDS–PAGE using a 5% stacking gel and a 10% separating gel. The sample and protein molecular weight markers were diluted with loading buffer under reducing and nonreducing conditions (loading buffer with and without β-mercaptoethanol, respectively) and boiled in water for 5 min. Then, 20 μL of the prepared sample solution was loaded onto the SDS–PAGE. Electrophoresis was performed at a constant voltage of 80 V for 30 min in the stacking gel, followed by 120 V for 60 min in the separating gel. The separating gel was stained with 0.25% Coomassie Brilliant Blue staining solution. To further verify the glycoprotein nature of CTSP-W2 and assess the presence or absence of free polysaccharides or proteins, periodic acid-Schiff (PAS) staining was performed according to a previously reported method [20].

### 2.4. Chemical Composition and Molecular Weight Determination

The total sugar content was determined using the phenol-sulfuric acid method. The absorbance values of glucose standards at different concentrations and those of the samples were measured at 490 nm, and the total sugar content was calculated according to the standard curve.

The protein content of CTSP-W2 was determined using a BCA kit and measured with a microplate reader. The molecular weight distribution of CTSP-W2 was analyzed using HPGPC in conjunction with a high-performance liquid chromatography system (LC-10A) equipped with a SUGAR BRT 105-103-101 column (8 mm × 250 mm; exclusion range = 0.1–1000 kDa), as described previously [21]. The column was eluted with 0.05 M NaCl at a flow rate of 0.8 mL/min, and detection was performed using the RI-10A refractive index detector. A calibration curve was constructed using dextran standards with molecular weights of 1153, 5000, 11,600, 23,800, 48,600, 80,900, 148,000, 273,000, 409,800, 667,800, and 3693,000 Da. Polysaccharide samples (5 mg) were dissolved in the mobile phase and filtered through a 0.22 μm membrane before further analysis.

The monosaccharide composition of CTSP-W2 was determined via ion chromatography (IC; ICS5000, Thermo Fisher Scientific, Waltham, MA, USA) using a Dionex Carbopac^TM^ PA20 column (3 mm × 150 mm) with electrochemical detection. The column was operated at 30 °C with a flow rate of 0.3 mL/min and an injection volume of 25 µL. Sixteen monosaccharide standards and glycoprotein samples were hydrolyzed with 3 M trifluoroacetic acid (TFA), dried under a nitrogen stream, and re-dissolved in deionized water for IC analysis. Absolute quantification was used to determine the monosaccharide content and molar ratios.

The amino acid composition of the glycoprotein was analyzed via ion chromatography (ICS5000, Thermo Fisher Scientific, Waltham, MA, USA) equipped with a Dionex AminoPac^TM^ PA10 column (2 mm × 50 mm) and electrochemical detection. The column was maintained at 30 °C, with a flow rate of 0.25 mL/min and an injection volume of 25 µL. Briefly, 5 mg of the sample was placed in a sealed ampoule and hydrolyzed with 2 mL of 6 M hydrochloric acid at 100 °C for 8 h. After hydrolysis, the sample was dried under nitrogen, reconstituted in 5 mL of deionized water, and diluted by mixing 100 µL of the solution with 900 µL of deionized water. The mixture was centrifuged at 12,000 rpm for 5 min, and the supernatant was subjected to IC analysis.

### 2.5. Structural Analysis of CTSP-W2

#### 2.5.1. Ultraviolet and Fourier Transform Infrared Spectra

Ultraviolet–visible (UV-Vis) spectra were recorded using a UV spectrophotometer (P9, Mapada, Shanghai, China). Infrared spectra were collected over the wavelength range of 400–4000 cm^−1^ using a Fourier transform infrared (FT-IR) spectrophotometer (IRAffinity-1, Shimadzu, Kyoto, Japan).

#### 2.5.2. Methylation Analysis

Following the method of Taylor R. L. and Needs [22], the carboxyl groups of CTSP-W2 were first reduced. Subsequently, CTSP-W2 (2–3 mg) was methylated three times using powdered NaOH in a DMSO-MeI system. The disappearance of the absorption band at 3700–3200 cm^−1^ corresponding to hydroxyl groups in the FT-IR spectrum indicated the complete methylation of CTSP-W2.

The methylated products were hydrolyzed with 2 M TFA (1 mL) at 100 °C for 8 h and then reduced with NaBH_4_. After adjusting the pH to neutral with 0.1 M acetic acid, the solution was acetylated with equivalent amounts of acetic anhydride and pyridine at 100 °C for 1 h. The resulting products were then examined using gas chromatography-mass spectrometry (GC–MS), performed using a Trace 1300 gas chromatograph coupled with an ISQ 7000 mass spectrometer (Thermo Scientific, Waltham, MA, USA). The GC-MS conditions were set as follows: an HP-INNOWAX capillary column (30 m × 0.32 mm × 0.25 µm; oven temperature program starting at 140 °C and increasing to 230 °C at a rate of 1 °C/min; inlet temperature of 250 °C; detector temperature of 250 °C; helium as the carrier gas at a flow rate of 1 mL/min.

#### 2.5.3. Nuclear Magnetic Resonance Analysis

Thirty-five milligrams of CTSP-W2 were dissolved in 600 μL of D_2_O (58.3 mg/mL) using 3-(trimethylsilyl)-1-propanesulfonic acid sodium salt as the internal standard. Both one-dimensional (1D) and two-dimensional (2D) nuclear magnetic resonance (NMR), including ^1^H NMR, ^13^C NMR, COSY, HSQC, NOESY, and HMBC spectra were collected over 48 h at 40 °C using a Bruker Avance III 600 NMR spectrometer (Beijing, China). The NMR spectra were analyzed using MestReNova software (version 5.3.1-4696, 2009, Mestrelab Research S.L., Santiago, Spain).

#### 2.5.4. Scanning Electron Microscopy Analysis

A thin layer of gold was applied to the glycoprotein sample using a sputter coater, and the morphology was assessed with a Hitachi S-4800 scanning electron microscope (Hitachi High-Technologies Corporation, Tokyo, Japan) operating at 10 kV under various magnifications.

#### 2.5.5. β-Elimination Reaction

Five milligrams of glycoprotein was dissolved separately in 5 mL of 0.2 M NaOH solution and 5 mL of distilled water, and the solutions were then heated at 45 °C for 3 h. The UV absorption spectra of the two solutions were recorded over the range of 190–400 nm. The glycopeptide bonding pattern was inferred from changes in UV absorption after the β-elimination process.

#### 2.5.6. Circular Dichroism Analysis

CTSP-W2 (3.0 mg) was dissolved in ultrapure water to prepare a 1.0 mg/mL solution. Circular dichroism (CD) spectra were recorded at room temperature using a JASCO CD spectrometer (JASCO Corporation, Hachioji, Tokyo, Japan) with a quartz cuvette of 1 mm (0.1 cm) path length. The spectra were collected over the wavelength range of 180–280 nm at a scan rate of 100 nm/min, with ultrapure water as the blank control.

#### 2.5.7. Congo Red Test

A 1 mL aliquot of the sample solution was mixed with an equal volume of 100 μM Congo red solution (1:1, *v*/*v*). Different concentrations of NaOH were then added to achieve final concentrations of 0, 0.1, 0.2, 0.3, 0.4, 0.5, 0.6, and 0.7 M. The mixtures were incubated for 10 min at 25 °C in the dark. A solution without a sample was prepared as a control. UV-Vis absorption spectra were measured over the wavelength range of 200–800 nm using a UV spectrophotometer (P9, Mapada, Shanghai, China) to determine the maximum absorption wavelength.

### 2.6. Immunomodulatory Activity of CTSP-W2

#### 2.6.1. Cell Culture

RAW 264.7 mouse macrophages (Cat. No. SCSP-5036) were purchased from the Shanghai Cell Bank, Chinese Academy of Sciences, and cultured in high-glucose DMEM supplemented with 10% FBS at 37 °C in a humidified atmosphere containing 5% CO_2_.

#### 2.6.2. Cell Viability and Phagocytic Activity in RAW 264.7 Cells

Cell cytotoxicity of CTSP-W2 was evaluated using the CCK-8 assay, following a previous report [23]. RAW 264.7 cells were cultured in DMEM supplemented with 10% FBS at 37 °C in a humidified incubator containing 5% CO_2_ until the logarithmic growth phase was attained. Cells were gently detached from the culture flask using a cell scraper, resuspended at a concentration of 1 × 10^5^ cells/mL, and inoculated into 96-well plates at 100 μL/well.

The cells were allowed to adhere for 24 h at 37 °C with CO_2_ incubator (5% CO_2_). For the experimental groups, CTSP-W2 was added at final concentrations of 25, 50, 100, 200, and 400 μg/mL. LPS (2.5 μg/mL) was used as a positive control. After 24 h of incubation, the culture supernatants were removed, and 100 µL of medium containing 10% CCK-8 reagent was added to each well. Plates were incubated at 37 °C for 2 h, and the absorbance (OD value) at 450 nm was measured using a microplate reader (1510, Thermo Fisher Scientific, USA). Each condition was tested in triplicate. Cell viability was calculated using the following formula:

$$Cell\;viability\left(\%\right)=\frac{A1-A0}{A2-A0}\times 100\%,$$

where A1 is the absorbance of the experimental group (containing cell culture medium, CCK-8, and polysaccharide solution), A2 is the absorbance of the control group (containing cell culture medium, CCK-8, and without polysaccharide), and A0 is the absorbance of the blank/negative group (containing cell culture medium, CCK-8, and without cells or polysaccharide).

Phagocytic activity of CTSP-W2 was assessed using the neutral red uptake method, following a previous report [24]. RAW 264.7 cells were seeded into 96-well plates at a density of 1 × 10^5^ cells/mL (100 μL/well) and cultured in a humidified CO_2_ incubator (37 °C, 5% CO_2_). After cell adhesion, cells were treated with CTSP-W2 glycoprotein at different concentrations (25, 50, 100, and 200 μg/mL) or LPS (2.5 μg/mL) for 24 h. Cells cultured in complete DMEM medium without CTSP-W2 or LPS treatment were used as the negative control group. After treatment, cells were washed twice with phosphate-buffered saline (PBS), and 100 µL of medium containing 10 µL of neutral red staining solution was added to each well. The plates were incubated for 2 h at 37 °C. At the end of incubation, the staining solution was removed, and the cells were washed twice with PBS. Subsequently, 100 µL of neutral red lysate was added to each well, and the plates were gently shaken on a shaker at room temperature for 10 min to ensure adequate lysis. The absorbance of each well was measured at 540 nm using a microplate reader. The phagocytosis index (PI) was calculated as follows:

$$PI\left(\%\right)=\frac{A1}{A2}\times 100\%,$$

where A1 is the absorbance of the experimental group, and A2 is the absorbance of the control group (polysaccharides-free solution).

#### 2.6.3. NO Production and Cytokine Secretion of RAW 264.7 Cells

RAW 264.7 cells were seeded into 24-well plates at a density of 1 × 10^6^ cells/well and treated with CTSP-W2 glycoprotein at concentrations of 100 and 200 μg/mL for 24 h. Cells cultured in medium alone served as the blank/negative control, while cells treated with 2.5 μg/mL LPS served as the positive control. The level of NO in the culture supernatant was measured using the Griess method [25]. The levels of IL-6, TNF-α, andIL-1β in the supernatant were quantified using precoated mouse ELISA kits (Dakewei Bioengineering Co., Ltd., Shenzhen, China) following the manufacturer’s instructions.

### 2.7. TNF-α, IL-6, and NO Levels After Inhibiting TLRs

RAW 264.7 cells were seeded into 24-well plates at a density of 1 × 10^5^ cells/mL (1 mL per well) and cultured for 24 h at 37 °C in a humidified incubator with 5% CO_2_ to allow cell adhesion. The cells were then pretreated with the TLR antagonists C29 (50 µM) for 1 h, TAK-242 (100 nM) for 4 h, or E6446 (30 nM) for 1 h, as described previously [26,27,28]. Following pretreatment, the cells were stimulated with CTSP-W2 (100 or 200 µg/mL). Cells treated with 2.5 µg/mL lipopolysaccharide (LPS) served as the positive control, while cells cultured in medium alone served as the negative control. After 24 h of incubation at 37 °C in a humidified atmosphere containing 5% CO_2_, the culture supernatants were collected. The concentrations of TNF-α, IL-6, and nitric oxide (NO) in the supernatants were determined using commercially available ELISA kits and an NO detection kit according to the manufacturers’ instructions.

### 2.8. Western Blot Analysis

RAW 264.7cells (3 × 10^6^ cells/well) were seeded in 6-well plates and cultured for 12 h and treated as described in Section 2.6.2. The cells were incubated with CTSP-W2 glycoprotein at concentrations of 100 and 200 μg/mL or with LPS (2.5 µg/mL) and medium alone served as the blank/negative control for 24 h. After treatment, the cells were lysed on ice for 30 min using Western and immunoprecipitation (IP) lysis buffer (strong RIPA lysis buffer, Proteintech) supplemented with 1 mM phenylmethylsulfonyl fluoride (PMSF). Protein concentrations were determined using a BCA protein detection kit. Protein samples (20 μg per lane) were separated via 10% SDS-PAGE and transferred to polyvinylidene fluoride (PVDF) membranes. Then, PVDF membranes were blocked with 5% BSA for 1 h at room temperature with gentle shaking and incubated overnight at 4 °C with primary antibodies (from rabbit polyclonal/monoclonal antibodies) against TLR4, TLR9, P-p65, p65, P-p38, p38, P-IKBα, P-IKBα, and β-actin. After three washes with TBST, the membranes were incubated with appropriate secondary antibodies for 1 h at room temperature. Protein bands were visualized using Super ECL Plus solution and captured using a Tanon-5200 automatic chemiluminescence imaging analysis system (Tanon Science & Technology Co., Ltd., Shanghai, China), and the grayscale intensity of target protein bands was quantified using ImageJ software (1.54p version).

### 2.9. Molecular Docking

Molecular docking was performed to analyze interactions between CTSP-W2 and TLR proteins. The CTSP-W2 ligand was constructed using the GLYCAM-Web carbohydrate builder (Complex Carbohydrate Research Center, University of Georgia), and the crystal structures of TLR2 and TLR4 proteins were downloaded from the RCSB PDB database (https://www.rcsb.org/, accessed on 12 November 2025). Protein–carbohydrate docking simulations were conducted using the HDOCK server (http://hdock.phys.hust.edu.cn/, accessed on 18 November 2025). The receptor (TLR4/MD-2 and TLR2) and the ligand (CTSP-W2) were prepared in PDB format. A blind docking strategy was adopted, with no binding pocket constraints defined, and all remaining docking parameters were maintained at default values. Hydrogen bonds, which play a significant role in the structure and function of biological molecules, were used as the basis for analyzing ligand–receptor interactions.

### 2.10. Statistical Analysis

Experimental data are expressed as mean ± standard deviation and analyzed using GraphPad Prism 10 software. Statistical significance was determined using two-way analysis of variance (ANOVA) followed by Dunnett’s multiple comparisons test. Differences were considered statistically significant at *p* < 0.05, *p* < 0.01, and *p* < 0.001 compared to the control group.

## 3. Results

### 3.1. Separation and Purification of CTSP-W2

**Figure 1:**
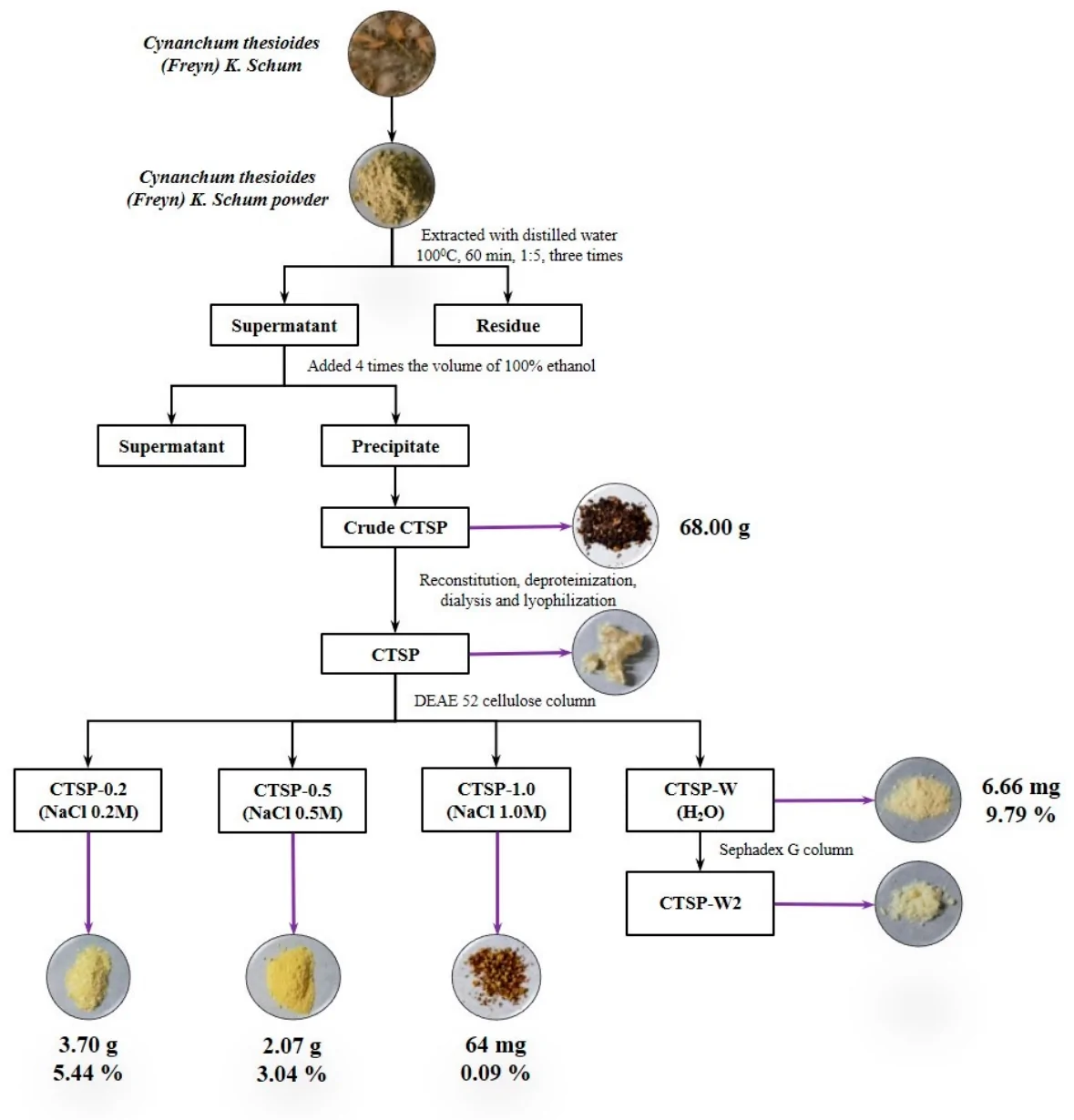
Extraction and purification flow diagram of CTSP-W2.

**Figure 2:**
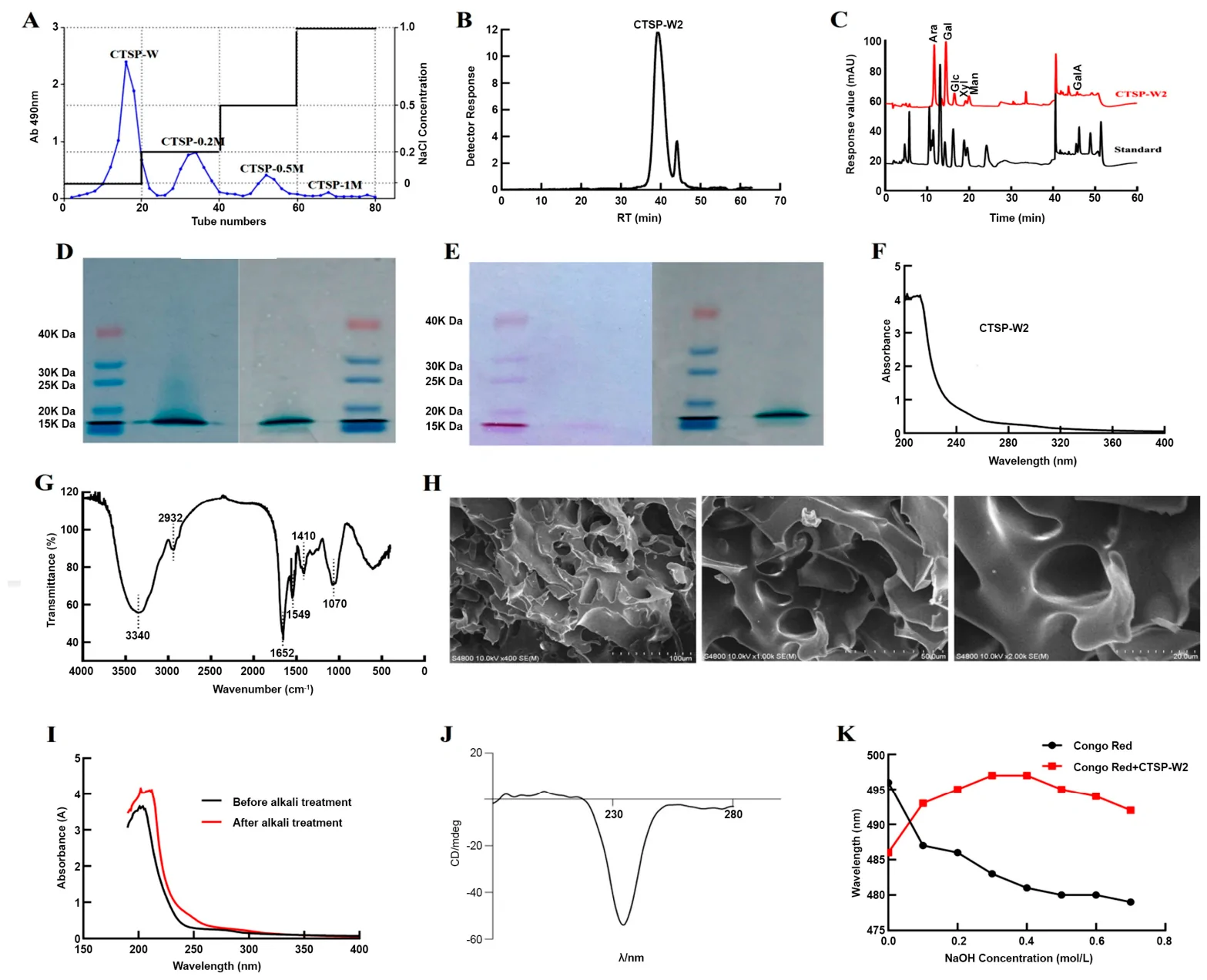
Structural characterization and physicochemical analysis of CTSP-W2. (**A**) Elution profile of CTSP-W2 from DEAE-52 anion exchange chromatography. (**B**) Molecular weight distribution determined via HPGPC. (**C**) Monosaccharide composition determined via ion chromatography. (**D**) SDS–PAGE electropherogram of CTSP-W2 stained with Coomassie brilliant blue under nonreducing (left) and reducing conditions(right); lanes: M, molecular weight marker. (**E**) SDS-PAGE electropherogram of CTSP-W2 stained with PAS (left) and Coomassie brilliant blue (right); lanes: M, molecular weight marker. (**F**) UV spectrum of CTSP-W2. (**G**) FT-IR spectrum of CTSP-W2. (**H**) SEM images of CTSP-W2 at ×400, ×1000, and ×2000 magnifications, revealing its porous network morphology. (**I**) UV spectrum after β-elimination treatment, indicating the glycoprotein linkage type. (**J**) CD spectrum of CTSP-W2. (**K**) Congo red assay showing conformational changes in CTSP-W2 under different NaOH concentrations.

The crude glycoprotein (CTSP) was extracted from *C. thesioides* through hot water extraction followed by alcohol precipitation (Figure 1). The extraction rate, total sugar content, and protein content of the crude extract were 10.28% ± 0.45%, 33.16% ± 0.27%, and 12.30% ± 0.13%, respectively. DEAE-52 anion-exchange columns are commonly used to separate polysaccharides based on their charge: fractions eluted with distilled water are typically neutral polysaccharides, whereas fractions eluted with NaOH solutions of different concentrations exhibit acidic properties [29]. 68.00 g of CTSP was further fractionated using anion-exchange chromatography with DEAE cellulose-52. Four elution peaks, namely, CTSP-W, CTSP-0.2M, CTSP-0.5M, and CTSP-1.0M, were detected during gradient elution with 0, 0.2, 0.5, and 1.0 M NaCl, with respective yields of 9.79%, 5.44%, 3.04% and 0.09% (Figure 2A). The major fraction, CTSP-W, was further purified on a Sephadex G-100 gel column, yielding three fractions collected at different elution times (37–54.5, 63–83, and 91–111 min; Figure S1). HPGPC analysis revealed that the second fraction (63–83 min) exhibited a single symmetrical peak and was designated CTSP-W2, with a yield of 12.1% (Figure 2B). The average molecular mass of CTSP-W2 was 9.862 × 10^3^ Da, calculated using dextran standards and the calibration curve of logM_w_ versus retention time (*y* = −0.1948*x* + 11.649, *R*^2^ = 0.9947).

### 3.2. General Chemical Analysis of CTSP-W2

Monosaccharide composition analysis is crucial in glycoproteins studies because their biological activities and functional properties are closely related to the types, ratios, and linkages of monosaccharide species. As shown in Figure 2C, CTSP-W2 is composed of galactose, arabinose, glucose, galacturonic acid, and xylose, with a molar ratio of 0.498:0.274:0.086:0.053:0.052:0.037. Galactose is the predominant component, followed by arabinose and glucose. The low content of galacturonic acid indicates that CTSP-W2 is a neutral glycoprotein. Amino acid analysis showed that CTSP-W2 contains 6.83% protein, comprising 18 amino acids. Among these, asparagine was the most abundant (108.81 µg/mg), while glutamine, glutamic acid, arginine, lysine, glycine, and cysteine were also relatively high. Seven essential amino acids were detected (Table 1). These results suggest that CTSP-W2 has a rich amino acid composition, mainly comprising nonaromatic amino acids, with particularly high levels of asparagine, glutamine, and glutamic acid, which may be closely related to its potential functional properties. Asparagine is a nonessential amino acid that plays an important role in nitrogen metabolism and the synthesis of proteins and nucleic acids [30]. It has been reported to relieve fatigue and enhance stamina, demonstrating its potential nutritional and functional value [31]. As depicted in Figure 2D and Figure S2, CTSP-W2 showed a single band on SDS-PAGE under reducing (left) and nonreducing (right) conditions, with a molecular mass of ~15 kDa, determined by Coomassie brilliant blue staining using a protein marker. Meanwhile, PAS staining of SDS–PAGE gels revealed a single band at the same position (Figure 2E). These results demonstrate that CTSP-W2 is a monomeric protein without any disulfide-bonded subunit and is homogenous, containing no free protein or polysaccharide. Lu et al. identified the glycoprotein SGP1 from *Sipunculusnudus* using the same method and found that SGP1 had an approximate molecular weight of 9.26 kDa and a monomeric nature, as determined via reduced and nonreduced SDS-PAGE [32]. Notably, the average molecular weight of CTSP-W2 measured via HPGPC was lower than that determined through SDS-PAGE. This discrepancy is likely due to glycosylation, which can alter the conformation of SDS-bound proteins or affect their migration. It has been reported that the apparent molecular weight of glycoproteins in SDS–PAGE may be higher than their actual molecular weight [32]. Glycosylation reduces the binding of SDS to proteins and increases their Stokes radius, resulting in slow migration and a high apparent molecular weight.

### 3.3. Structural Analysis of CTSP-W2

#### 3.3.1. UV and FT-IR Spectra

The UV spectrum of CTSP-W2 (Figure 2F) showed no obvious absorption peaks between 260 and 280 nm, indicating a low content of aromatic amino acids and the absence of nucleic acids. This result is consistent with the amino acid composition analysis [32]. The FT-IR spectrum of CTSP-W2 (Figure 2G) exhibited characteristic absorption peaks corresponding to functional groups in polysaccharide and protein structures. A broad peak at ~3340 cm^−1^ corresponded to the overlapping stretching vibrations of O-H and N-H bonds, indicating hydrogen bonds from polysaccharide or protein molecules [33]. The absorption peaks at 2932 and 1410 cm^−1^ were attributable to the stretching and bending vibrations of C-H bonds. The strong absorption peaks at 1652 cm^−1^ corresponded to the C=O stretching vibration of carboxyl groups from polysaccharides and amino acids such as asparagine. Another characteristic absorption peak at 1549 cm^−1^ arose from the stretching vibration of N-H or C-N bonds in amino acids [34]. Moderate absorption peaks in the range of 1000–1200 cm^−1^ were related to the stretching vibrations of C-O-H and C-O-C bonds, with the peak at 1070 cm^−1^ likely due to the stretching vibrations of C-O-C bonds in the sugar ring.

#### 3.3.2. Methylation Analysis

The glycosidic linkage types of CTSP-W2 were further elucidated via methylation and GC–MS (Table 2). Monosaccharide analysis revealed that the uronic groups of galacturonic acid were first reduced, followed by methylation, hydrolysis, and acetylation. CTSP-W2 comprised 16 complex sugar residues, among which the molar ratios of Gal and Ara residues were the highest, at 0.531 and 0.258, respectively, consistent with the results of the monosaccharide composition analysis. As the predominant monosaccharide in CTSP-W2, Gal residues included seven types of glycosidic bonds: →3,6)-Galp-(1→, →6)-Galp-(1→, →3)-Galp-(1→, Galp-(1→, →4,6)-Galp-(1→, →4)-Galp-(1→, →2)-Galp-(1→, with molar ratios of 0.137:0.118:0.081:0.078:0.043:0.043:0.031, respectively. The other main sugar residue, Ara, was associated with three types of glycosidic bonds: →5)-Araf-(1→, Araf-(1→, and →3,5)-Araf-(1→, with molar ratios of 0.121:0.115:0.022, respectively. These results suggest that CTSP-W2 possesses abundant linear chains and branched linkages.

#### 3.3.3. Scanning Electron Microscopy Analysis

The ultrastructural surface characteristics of the glycoprotein were observed using scanning electron microscopy (SEM) at magnifications of ×500, ×1000, and ×2000. SEM images of CTSP-W2 at ×500 (Figure 2G, left) revealed a porous structure with uneven pore sizes and wrinkled pore walls, resembling a complex three-dimensional (3D) network-like structure. This indicates a large specific surface area and a spatially crosslinked or entangled molecular structure, often formed by long-chain polysaccharide backbones with branched chains and hydrogen bonding [5]. At ×2000 magnification (Figure 2G, right), the surface microstructure exhibited layered superposition, with dendritic structures becoming more apparent on the surface. The porous and irregular surface provides a high specific surface area and enhanced adsorption properties, which may promote substance delivery, free radical trapping, and interactions with biomolecules, thus potentially enhancing antioxidant, immunomodulatory, and anti-inflammatory activities [35]. In addition, the 3D network structure of CTSP-W2 confers mechanical stability, supporting its functional performance in complex biological environments [7]. These structural properties provide an important physical basis for the biological activities of CTSP-W2.

#### 3.3.4. β-Elimination Reaction

In dilute alkaline solutions, O-glycosidic bonds linking sugars to the hydroxyl groups of Ser or Thr are susceptible to breakage and are subsequently converted into α-aminoacrylic and α-aminobutenic acids, which show a characteristic absorption at 240 nm. By contrast, N-glycosidic bonds remain stable under basic conditions [36]. To further analyze the glycopeptide chains for the presence of N- and O-linked sugar chains, the UV spectrum of CTSP-W2 after alkali treatment was recorded over the range of 190–400 nm using a UV spectrophotometer. As shown in Figure 2I, the UV absorptions of CTSP-W2 increased significantly at 240 nm following treatment with 0.2 M NaOH, indicating the existence of a β-elimination reaction and confirming the presence of O-glycosidic bonds in CTSP-W2.

#### 3.3.5. Circular Dichroism Analysis

We employed circular dichroism (CD) spectroscopy to analyze the secondary structure of CTSP-W2, which contain numerous chromophores that produce CD signals. In the far-Uv region (180–240 nm), corresponding to the peptide bond absorption region, CD spectra can be used to determine the secondary structure characteristics, such as the content of α-helices and β-folds [37]. The CD spectrum of the glycoprotein revealed distinct structural attributes. As shown in Figure 2J, the far-UV CD spectrum of CTSP-W2 exhibits a strong negative Cotton effect near 234 nm and a weak positive Cotton effect near 190 and 203 nm. The negative peak at 234 nm, indicative of disulfide bonds (cysteine), signifies the presence of an abundant and potentially well-defined disulfide bond network. The spectral features below 230 nm suggest a predominantly disordered structure with a notably low content of ordered secondary elements. The near-UV CD spectrum was flat, suggesting a stable and uniform tertiary environment with minimal contributions from aromatic amino acid side chains [38]. Collectively, these data indicate that CTSP-W2 is a disulfide-rich, chiral glycoprotein that adopts a stable, predominantly disordered conformation.

#### 3.3.6. Triple-Helix Structure Analysis

Congo red, an anionic dye, forms complexes with triple-helical polysaccharides. Increasing the concentration of NaOH disrupts these polysaccharide–Congo red complexes, resulting in a redshift of its maximum absorption wavelength. The mixture containing CTSP-W2 showed a significant redshift of the maximum absorption wavelength at NaOH concentrations of 0.1–0.4 M (Figure 2K), suggesting that the polysaccharide possesses a stable triple-helical spatial conformation. No similar changes were observed in the control group [39]. With further increase in the NaOH concentration, the maximum absorption wavelength of the mixture showed a slight blueshift, suggesting that the triple-helical structure was gradually disrupted under strongly alkaline conditions, reflecting limited structural stability. Previous studies have demonstrated that the conformation of glycoproteins is closely associated with their biological activity [32]. Therefore, it is hypothesized that the triple-helix structure of CTSP-W2 may enhance its biological activity. These findings are consistent with those obtained from CD spectroscopy.

#### 3.3.7. NMR Analysis

**Figure 3:**
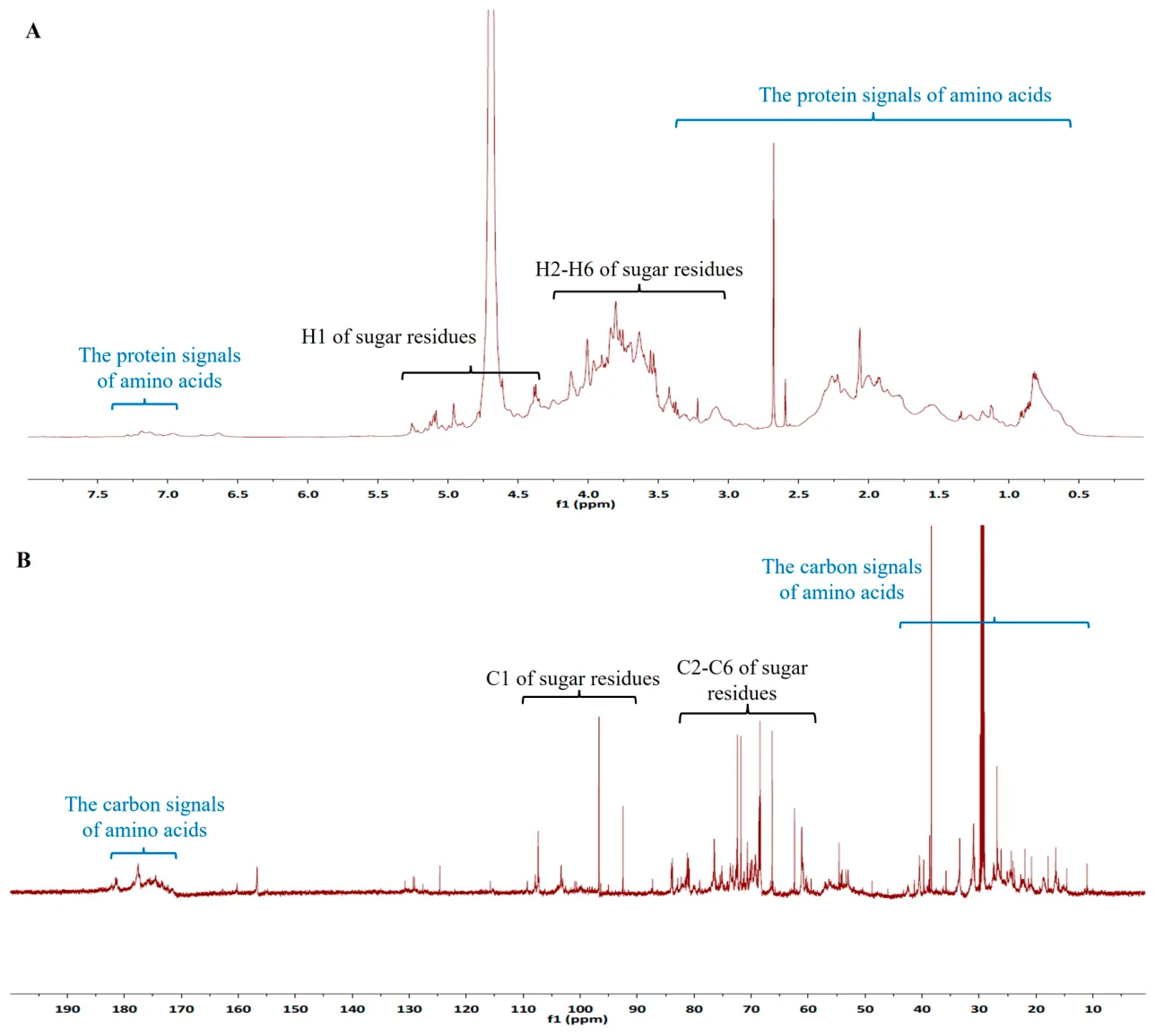

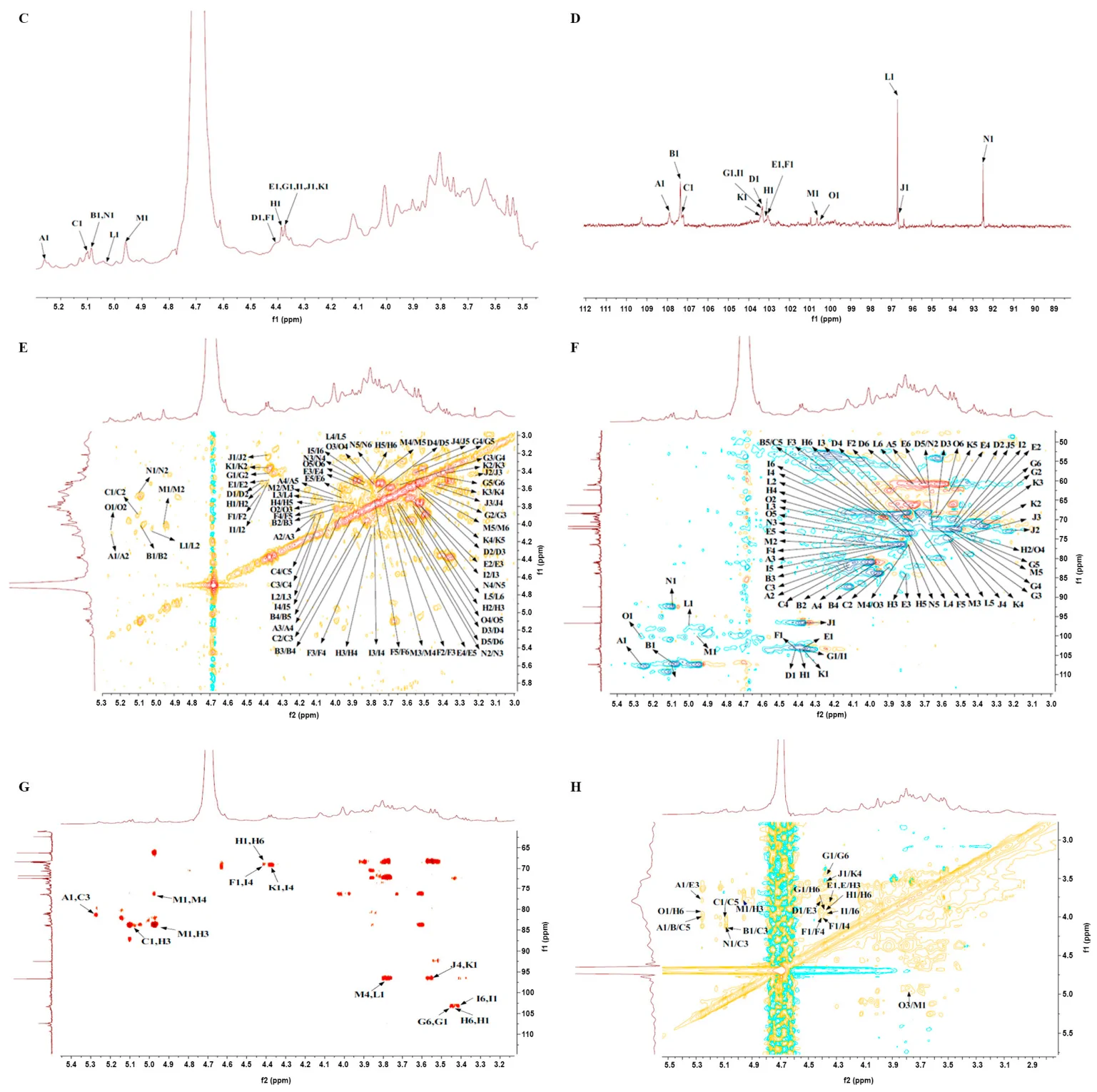

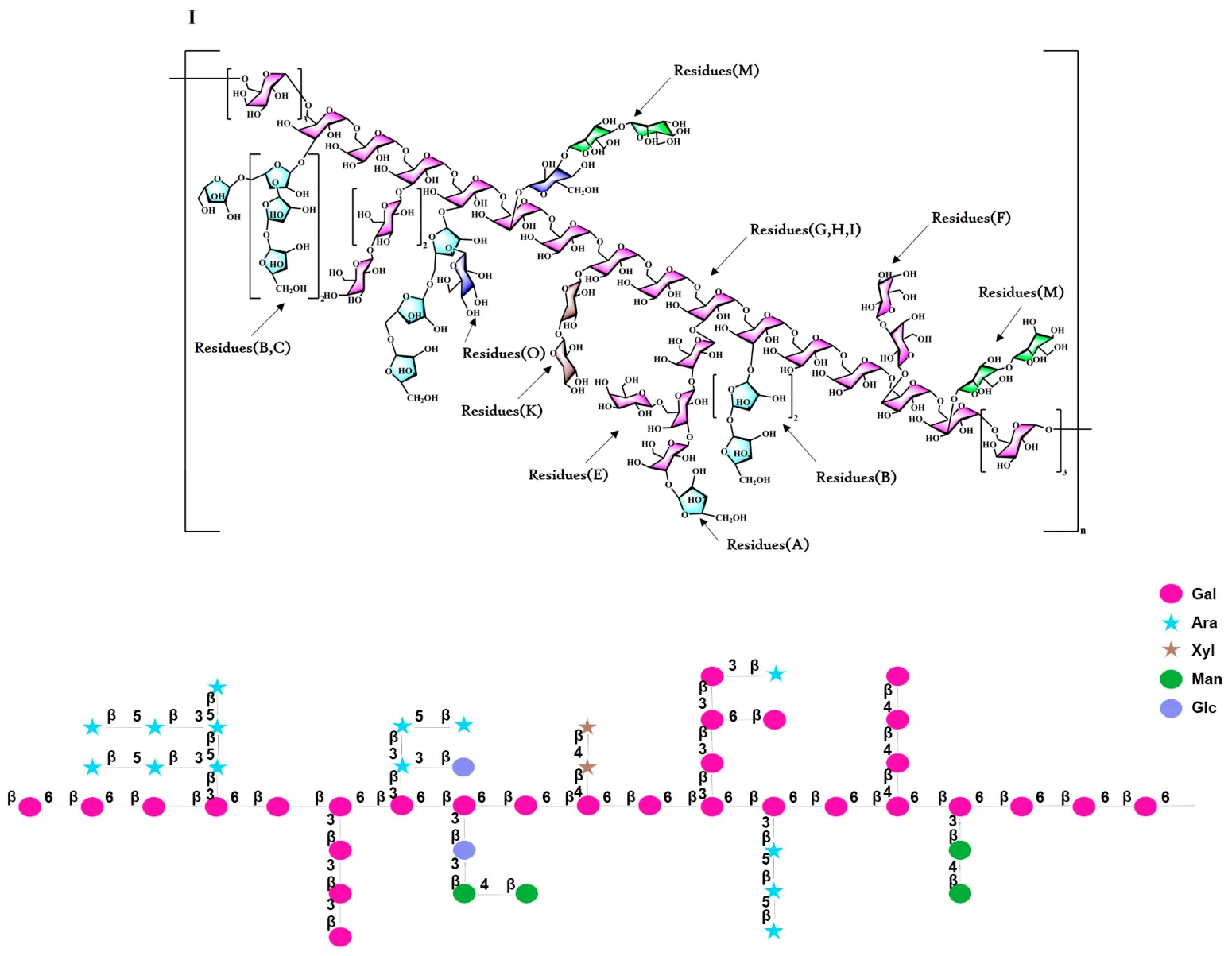
NMR characterization of the CTSP-W2 glycoprotein. (**A**) ^1^H NMR spectra (0–8 ppm), (**B**) ^13^C NMR spectra (0–200 ppm), (**C**) ^1^H NMR spectra (3.4–5.3 ppm), (**D**) ^13^C NMR spectra (88–112 ppm), (**E**) COSY spectra, (**F**) HSQC spectra, (**G**) HMBC spectra, (**H**) NOESY spectra, and (**I**) proposed structural fragment of CTSP-W2.

The primary structure of CTSP-W2 was further investigated using NMR techniques, namely, 1D (^1^H NMR and ^13^C NMR) and 2D NMR spectra such as HSQC, HMBC, COSY, and NOESY. In addition to the characteristic signals of sugar residues, the presence of typical proton signals from amino acids such as glutamic acid and valine at 1.0–3.5 ppm, as well as signals from histidine around 7.5–8.0 ppm in Figure 3A [40,41], combined with carbon signals in the regions of 10–40 ppm and around 175 ppm in Figure 3B, collectively indicate that the sample is a glycoprotein [42]. The different linkages of sugar residues were labeled as A-O; their corresponding chemical shifts are summarized in Table 3. The ^1^H NMR spectrum exhibited predominant proton signals between *δ* 3.0 and 5.3 ppm, with anomeric proton signals observed in the *δ* 4.3–5.5 ppm range (Figure 3C) [43]. The ^13^C NMR spectrum displayed clear signals, highlighting anomeric carbons between *δ* 90 and 110 ppm [44,45], suggesting the presence of 15 distinct sugar residues (Figure 3D). Additionally, a signal at *δ* 177.75 ppm indicated the presence of uronic acid, consistent with the FT-IR and monosaccharide composition analyses [46]. Typical signals in the δ 20–40 ppm range of the ^13^C NMR spectrum corresponded to amino acid residues of the protein moiety [41].

Based on methylation analysis and following 2D NMR spectra, four main sugar residues, Araf-(1→) (residue A), →5)-Araf-(1→ (residue B), →6)-Galp-(1→) (residue G), and →3,6)-Galp-(1→ (residue H), were identified using COSY (Figure 3E) and HSQC (Figure 3F) spectra. The homonuclear ^1^H–^1^H COSY spectra revealed correlations between neighboring protons within the sugar residues. The signal peaks A (*δ* 5.26/δ 4.11), B (*δ* 5.09/δ 4.01), G (*δ* 4.37/δ 3.43), and H (*δ* 4.39/δ 3.56) were assigned to the H1 and H2 coupled protons of Araf-(1→), →5)-Araf-(1→), →6)-Galp-(1→), and →3,6)-Galp-(1→), respectively. The chemical shifts in H1-H6 for all 15 monosaccharide residues were determined based on the coupling correlations of adjacent protons (Table 3).

Three strong anomeric carbon signals for Araf residues (A–C) were observed at *δ* 107.79–107.27 ppm, corresponding to crosslinked anomeric proton signals at 5.26–5.09 ppm in the HMQC spectra (Figure 3G) [47]. Anomeric signals in the *δ* 106–110 ppm region were likely assigned to β-Galp residues (D-I) in the same spectra [48]. The chemical shifts in C1–C6 for each of the 15 sugar residues were designated based on carbon-hydrogen coupling correlation (Table 3) [49,50].

The linkage patterns of the sugar residues in CTSP-W2 were further dissected by combining the HMBC (Figure 3E) and NOESY (Figure 3H) spectra. Cross-peaks observed at *δ* 5.26/81.09 ppm (A H1/C C3) and *δ* 5.10/83.91 ppm (C H1/E C3) indicated the presence of Araf-(1→), →3,5)-Araf-(1→, →3,6)-Galp-(1→ or →3)-Galp-(1→ linkages in the HMBC spectrum, while the corresponding signal in the NOESY spectrum appeared at *δ* 5.26/4.12 ppm (A H1/C H3). Cross-peaks observed at *δ* 4.96/83.91 ppm (M H1/H or E C3) and *δ* 4.96/76.41 ppm (M H1/M or F C4) indicated the presence of →4)-Manp-(1→, →3,6)-Galp-(1→ or →3)-Galp-(1→, and →4)-Manp-(1→, →4)-Manp-(1→ or →4)-Galp-(1→ linkages in HMBC (Figure 3G), while the corresponding NOSEY signals were observed at *δ* 4.96/3.80 ppm (M H1/H or E H3) and *δ* 4.96/4.10 ppm (M H1/M or F H4). Additionally, the cross-peaks observed at *δ* 3.78/96.70 ppm (M H4/L C1) indicated the presence of Manp-(1→, →4)-Manp-(1→ linkages in HMBC. Two cross signals observed at *δ* 3.44/103.63 ppm (G/I H6/G/I C1) and *δ* 3.41/103.39 (H H6/H C1) indicated the presence of →6)-Galp-(1→, →4,6)-Galp-(1→, and →3,6)-Galp-(1→ linkages in HMBC, with corresponding NOSEY signals at δ 4.36/3.45 ppm (G H6/G H1) and δ 4.38/3.92 ppm (H H6/H H1) (Figure 3H). Based on these results, a hypothetical structure for CTSP-W2 was proposed (Figure 3I).

The backbone of CTSP-W2 contained →6)-Galp-(1→ and →3,6)-Galp-(1→ with C3 branching to →5)-Araf-(1→, →3,5)-Araf-(1→, →4)-Manp-(1→, and 3)-Glcp-(1→. Subbranch linkage patterns are further illustrated in Figure 3E. Interestingly, additional cross-signals at *δ* 4.41/3.81 ppm (D H1/E H3), *δ* 4.41/3.95 ppm (F H1/I H4) and *δ* 4.97/3.78 ppm (M H1/O H3) observed in NOESY (Figure 3F) may indicate ^1^H–^1^H spatial correlations rather than ^13^C-^1^H linkage correlations, supporting the presence of a triple-helix structure in the glycoprotein.

### 3.4. CTSP-W2-Activated Macrophage Cells

#### 3.4.1. Cell Viability in RAW 264.7 Cells

**Figure 4:**
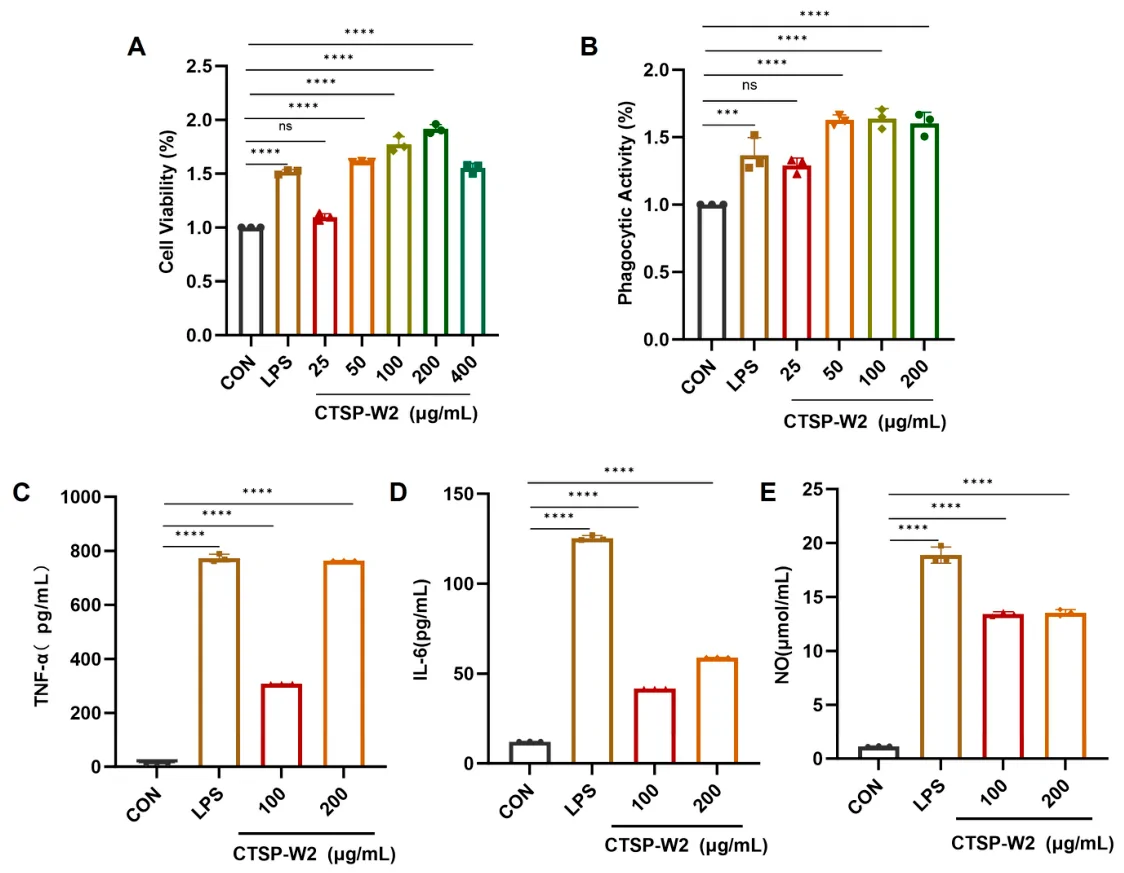
Immunomodulatory activity of CTSP-W2 glycoprotein on RAW 264.7 cells. RAW 264.7 cells were cultured with CTSP-W2 at different concentrations for 24 h. (**A**) Macrophage proliferation activity. (**B**) Phagocytic activity. The culture supernatants were assayed for the levels of (**C**) TNF-α, (**D**) IL-6, and (**E**) NO secretion. LPS was used as a positive control, *** *p* < 0.001 and **** *p* < 0.0001 versus the cell control. ns means no statistically significant difference.

Macrophages are pivotal effector cells in the immune system, playing essential roles in cellular and molecular immunity. They contribute critically to immune defense, immune regulation, and inflammatory responses [51]. The immunomodulatory potential of CTSP-W2 was evaluated by assessing its effects on RAW 264.7 macrophage viability using the CCK-8 assay. As shown in Figure 4A, treatment with LPS (2.5 μg/mL) significantly increased cell viability to ~151% (*p* < 0.001), confirming effective macrophage activation. Notably, CTSP-W2 treatment (25–400 μg/mL) further enhanced viability in a concentration-dependent manner. the tested concentrations (50–200 μg/mL) significantly exceeded the LPS group (*p* < 0.001), peaking at 50 μg/mL with 191%. Although viability slightly decreased at 400 μg/mL, it remained significantly higher than the blank control (*p* < 0.001).

#### 3.4.2. Phagocytic Activity of RAW 264.7 Cells

The effect of CTSP-W2 on phagocytic activity in RAW 264.7 macrophages was assessed using the neutral red uptake assay. As shown in Figure 4B, CTSP-W2 significantly enhanced phagocytosis across concentrations of 25–200 μg/mL compared to the blank control (*p* < 0.001), reaching 167% at 100 μg/mL. These results demonstrate that CTSP-W2 effectively enhances macrophage phagocytic capacity, indicating its immunomodulatory potential.

#### 3.4.3. NO Production and Cytokine Secretion of RAW 264.7 Cells

Macrophages are involved in innate immunity and inflammation, releasing inflammatory factors such as IL-6 and TNF-α during immune responses. IL-6 is an important pro-inflammatory cytokine, and its overexpression is associated with various inflammatory diseases [52]. TNF-α is a typical pro-inflammatory cytokine, and its elevated expression is often associated with inflammatory conditions [53]. As shown in Figure 4C,D, treatment with CTSP-W2 at concentrations of 100 and 200 µg/mL significantly increased the secretion of TNF-α and IL-6 in RAW 264.7 cells compared to the black/negative control group (*p* < 0.0001). Similarly, SGP1, a glycoprotein from *Sipunculus nudus*, exhibited significant immune-enhancing activity in RAW 264.7 macrophages by promoting cell proliferation, enhancing phagocytic capacity, and stimulating the secretions of NO, IL-1β, TNF-α, and IL-6 via NF-κB signaling pathways [32].

NO, synthesized by inducible NO synthase (iNOS), serves as a pivotal inflammatory mediator and signaling molecule. As shown in Figure 4E, NO production was significantly elevated following treatment with various concentrations of CTSP-W2 compared to the control (*p* < 0.0001), though levels remained lower than those observed with the positive control LPS, and no clear dose–response correlation was observed.

### 3.5. Molecular Mechanism of CTSP-W2-Activated Macrophages

#### 3.5.1. Effects of TLR on RAW 264.7 Cells

The innate immune response is essential for the survival of most organisms. Several cell types participate in this response, including dendritic cells, natural killer cells, macrophages, neutrophils, and eosinophils [54]. Immune cells express various pattern recognition receptors (PRRs), such as TLRs, C-type lectin receptors, RIG-I-like receptors, and NOD-like receptors. These receptors recognize pathogen-associated molecular patterns on foreign pathogens and endogenous damage-associated molecular patterns, thereby initiating immune responses that include the release of proinflammatory factors, interferons, and chemokines [51]. Among these, TLRs, particularly TLR2, TLR4, and TLR9, play vital roles in the molecular mechanisms underlying LPS-induced inflammation [9]. Studies have shown that natural polysaccharides can exert immunomodulatory effects by regulating TLR-related signaling pathways, modulating the expression of inflammatory factors in macrophages, and inhibiting excessive cytokine secretion [55].

**Figure 5:**
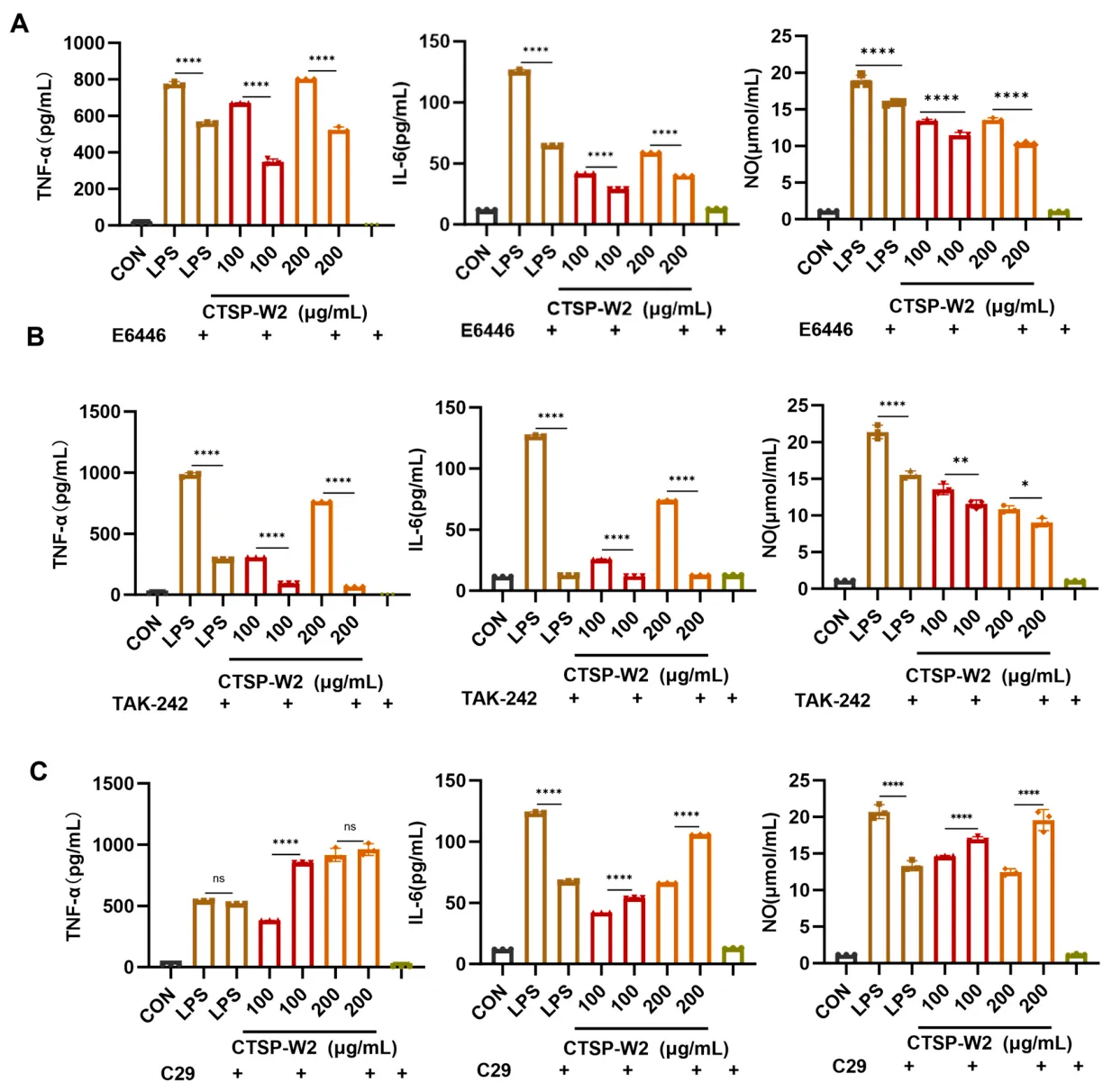
Expression levels of IL-6, TNF-α, ad NO in CTSP-W2-treated RAW 264.7 cells in the presence of three receptor inhibitors. (**A**) Levels of TNF-α, IL-6, and NO treated with anti-TLR9 (E6446). (**B**) Levels of TNF-α, IL-6, and NO treated with anti-TLR4 (TAK242). (**C**) Levels of TNF-α, IL-6, and NO treated with anti-TLR2 (C29). 100 and 200 are only CTSP-W2, 100+ and 200+ are CTPS-W2 at different concentrations added different receptor inhibitor, + mean only different receptor inhibitor. Each value represents the mean ± SD, *n* = 3. * *p* < 0.05, ** *p* < 0.01, and **** *p* < 0.001, ns means no statistically significant difference.

To further identify the specific receptors involved in activating RAW 264.7 cells through the NF-κB pathway, macrophages were treated with inhibitors of TLR4 (TAK-242), TLR2 (C29), and TLR9 (E6446) [56]. The effects of these inhibitors on the expression levels of IL-6, TNF-α, and NO in CTSP-W2-treated RAW 264.7 cells are shown in Figure 5. As shown in Figure 5A,B, the secretion of IL-6, TNF-α, and NO induced by LPS and CTSP-W2 was significantly suppressed in the presence of TLR4 and TLR9 inhibitors in a concentration-dependent manner compared to the inhibitor-free control (*p* < 0.0001). By contrast, the addition of the TLR2 inhibitor (C29) markedly upregulated TNF-α expression at 100 μg/mL (*p* < 0.0001), IL-6 expression at 100–200 μg/mL (*p* < 0.0001), and NO production at 200 μg/mL (*p* < 0.0001) (Figure 5C). These results indicate that the TLR signaling pathway plays a vital role in CTSP-W2-induced macrophage activation, with TLR4 and TLR9 serving as the major receptors mediating the macrophage interaction.

#### 3.5.2. Activation of TLR4/9-Mediated NF-κB and MAPK Signaling Pathways by CTSP-W2

The NF-κB and MAPK signaling pathways are interrelated key cascades that coordinately regulate immune responses. Both pathways are activated when pathogens are recognized by PRRs. NF-κB mainly controls the transcription of pro-inflammatory cytokines (e.g., TNF-α, IL-1β, and IL-6) and cell survival genes [57]. Simultaneously, the MAPK pathway (including the p38, JNK, and ERK subfamilies) regulates the activation of transcription factors such as AP-1, which further amplifies the expression of inflammatory genes and regulates cytokine stability, cell differentiation, and proliferation [58]. This synergistic crosstalk ensures a robust initial immune defense. Importantly, both pathways are subject to complex negative feedback mechanisms that prevent excessive inflammation, highlighting their important role in maintaining immune homeostasis. Dysregulation of these pathways is a hallmark of various inflammatory and autoimmune diseases.

**Figure 6:**
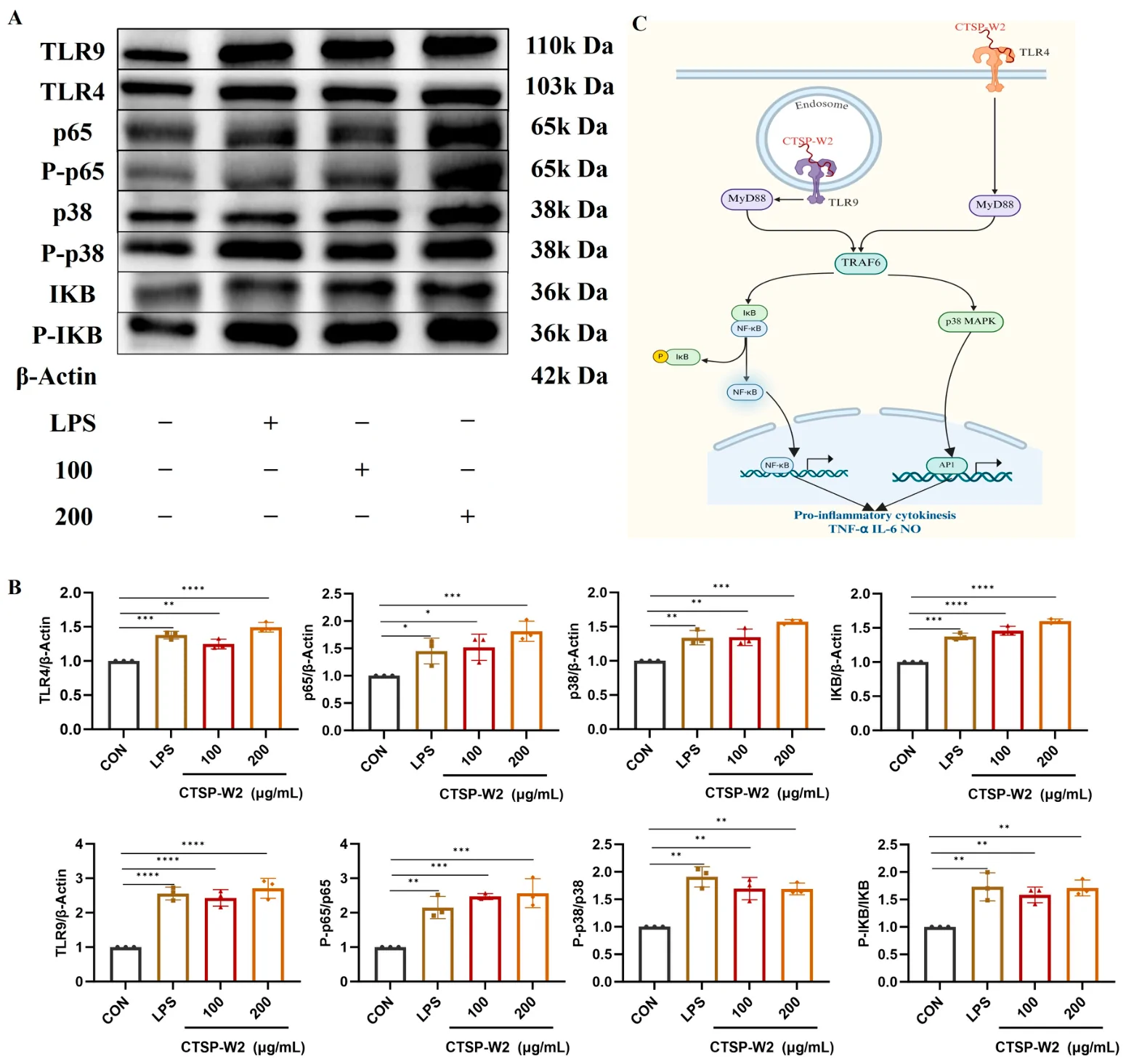
Effect of CTSP-W2 on RAW 264.7 cells activated via TLR4/9-mediated NF-κB and MAPK signaling pathways. RAW 264.7 cells were cultured in six-well plates and pretreated with CTSP-W2 (100 and 200 μg/mL) and LPS (2.5 μg/mL). After 12 h, total cellular protein was extracted, and the phosphorylation levels of MAPK (p38) and NF-κB (p65 and IκBα) were detected via Western blot. (**A**) Protein levels of TLR4, TLR9, p38, P-p38, p65, P-p65, IκBα, and P-IκBα analyzed via Western blot. (**B**) Quantification of TLR4, TLR9, p38/P-p38, P-p65/p65, and P-IκBα/IκBα levels. (**C**) Proposed mechanism through which CTSP-W2 modulates the activation of RAW 264.7 cells. Each value represents the mean ± SD, *n* = 3. * *p* < 0.05, ** *p* < 0.01, and *** *p* < 0.001 and **** *p* < 0.001, ns means no statistically significant difference.

To delineate the mechanism through which CTSP-W2 modulates the activation of RAW 264.7 cells, the protein expression levels of TLR4, TLR9, p38, P-p38, IκBα, P-IκBα, NF-κB p65, and phosphorylated NF-κB P-p65 were analyzed via Western blot (Figure 6A). As shown in Figure 6B, both LPS and CTSP-W2 treatments significantly upregulated the protein expression of TLR4 and TLR9 compared to the control group. Moreover, the CTSP-W2- group exhibited a higher level of statistical significance than the LPS group. Specifically, the upregulation of TLR4 expression was highly significant (*p* < 0.0001) in the CTSP-W2 group, whereas it was moderately significant (*p* < 0.001) in the LPS group. This TLR activation is associated with the subsequent stimulation of the downstream NF-κB and MAPK signaling pathways, suggesting that CTSP-W2 may exert its effect by binding to these receptors. Compared to the control, LPS and CTSP-W2 significantly upregulate the expression of P-p65/p65, P-p38/p38, and P-IκBα/IκBα (Figure 6A). The upregulation of IκBα and phosphorylated p65 proteins was more significant in the CTSP-W2 group (*p* < 0.0001 and *p* < 0.001, respectively) (Figure 6B). Upon CTSP-W2 stimulation, IκBα is phosphorylated and subsequently degraded, allowing the release and nuclear translocation of NF-κB p65. The phosphorylated NF-κB (P-p65) then acts as a transcription factor to promote the synthesis and release of various cytokines, thereby exerting immunomodulatory effects. These findings suggest that CTSP-W2 potentially mediates immunomodulation in RAW 264.7 cells by activating the TLR4/9-NF-κB-p38 MAPK signaling axis (Figure 6C).

### 3.6. Molecular Docking Analysis

**Figure 7:**
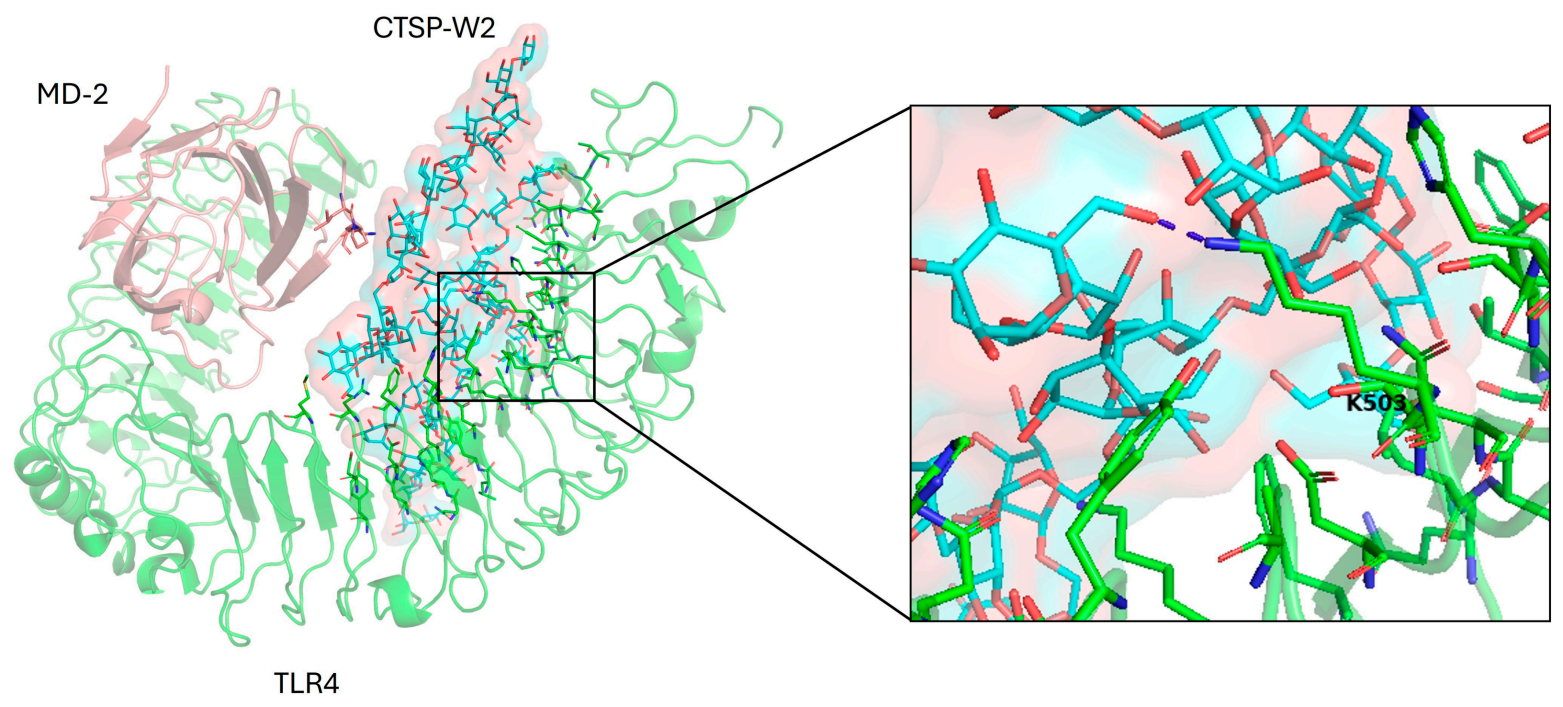
3D interaction diagram of CTSP-W2 with the TLR4/MD-2 complex.

One hundred docking conformations were generated using HDOCK, and the top 10 ranked models were selected for further evaluation. In the interaction between TLR4 and the proposed polysaccharide fragment of CTSP-W2, the predicted docking scores ranged from −624.2 to −476.1, with Model 1 achieving the most favorable docking score (−624.2) and the highest confidence score (0.9999) [59], indicating a highly probable binding mode (Table S1). Based on the molecular docking results of CTSP-W2 with the murine TLR4/MD-2 complex, the optimal conformation (Model 1) was selected for interaction analysis. The analysis revealed that CTSP-W2 primarily engages with the key residue LYS503 on chain A of TLR4 through multiple interactions, including two high-confidence hydrogen bonds and two hydrophobic contacts. Specifically, the O26 atom (Gal residue) and O13 atom (Man residue) of CTSP-W2 form hydrogen bonds with the terminal amino group (NZ) of the LYS503 side chain, with favorable distances of 1.6 and 2.56 Å, respectively. Among these, the O26-NZ distance of 1.6 Å indicates a strong hydrogen bond, which is critical for stabilizing the binding pose of the ligand (Figure 7). Additionally, the C47 and C48 atoms (Gal residue) within the polysaccharide backbone participate in hydrophobic interactions with the mid-chain carbon (CE) of LYS503, further reinforcing the binding interface. These results suggest that LYS503 in TLR-4 acts as a crucial interaction hotspot during CTSP-W2 binding, contributing both polar and nonpolar contacts that likely stabilize ligand association and may facilitate receptor activation. In the interaction between TLR2 and the proposed polysaccharide fragment of CTSP-W2, the predicted docking scores ranged from −483.1 to −438.6, with Model 1 achieving the most favorable docking score (−483.1) and the highest confidence score (0.9987) [59], (Table S2), indicating a lower probable binding mode compared to the TLR4 receptor. These results are consistent with findings from the specific inhibitor assay of TLRs.

## 4. Discussion

Although research on plant-derived glycoproteins is relatively recent, it has revealed their widespread distribution in plants. At least two classes of glycoproteins are present in plant cell walls: insoluble extensins and water-soluble, highly glycosylated arabinogalactan proteins (AGPs). Type II arabinogalactans (AGs) are found in various plant organs, including leaves, roots, stems, fruits, and flowers. Structurally, type II AGs are complex, highly branched polysaccharides characterized by a backbone of β-(1→3)-linked D-galactosyl residues, with side chains composed of β-(1→6)-linked D-galactosyl groups (Figure 4), as exemplified by larch AGs. These side chains may be further substituted with mono- or oligosaccharides, such as α-L-arabinofuranosyl or α-L-arabinopyranosyl residues, attached via (1→2), (1→3), (1→4), (1→5), or (1→6) linkages. Other substituents may include galactopyranose, arabinofuranose, arabinopyranose, and glucuronosyl residues from glucuronooligosaccharides [60,61].

Galactans are associated with extracellular spaces and plasma membranes. Compared to type I AGs, type II AGs exhibit a stronger association with proteins [62] and typically occur as carbohydrate moieties of extracellular proteoglycans known as AGPs, which are localized in plasma membranes and cell walls. Proteins bearing type II AGs represent minor components of the plant cell wall. Pollen contains substantial amounts of type II arabinogalactans, which have been implicated in immunomodulatory activities [63].

Herein, we isolated a homogeneous glycoprotein, designated CTSP-W2, using cellulose and gel column chromatography. Structural analysis revealed that CTSP-W2 consists of a (1→6)-linked galactan backbone (53.6%), with branches comprising arabinose (28.3%), mannose (10.6%), and glucose (3.83%) (Table 1). This glycoprotein contains a natural arabinogalactan structure featuring a β-(1→6)-linked D-galactosyl backbone, consistent with type II AGs.

M1-type macrophages, also known as classically activated macrophages, increase in proportion during inflammation and tissue injury and are characterized by high expression of pro-inflammatory cytokines, such as TNF-α, IL-6, and IL-1β. CTSP-W2 can activate various cell surface TLRs and the associated NF-κB signaling pathway, leading to the secretion of these cytokines. Studies have shown that polysaccharides with a high galactose content exhibit strong immunomodulatory activity [64,65], which may explain the immunomodulatory activity of CTSP-W2, as it contains large amounts of galactose. TLRs are key PRRs that initiate innate immunity. TLR2, located on the cell surface, forms heterodimers to sense bacterial lipoproteins. TLR4, in complex with MD-2, is the primary receptor for lipopolysaccharide, while intracellular TLR9 recognizes unmethylated CpG DNA.

Ligand binding typically triggers MyD88-dependent NF-κB activation, inducing the expression of inflammatory cytokines and bridging innate and adaptive immunity [9]. Western blot analysis and specific TLR inhibitor assays demonstrated that CTSP-W2 activates macrophages primarily via the TLR4/9 pathway rather than TLR2, subsequently mediating the NF-κB and MAPK signaling pathways (Figure 5 and Figure 6). As a positive control, although LPS does not directly activate TLR9, it can upregulate TLR9 expression through TLR4-triggered NF-κB and MAPK pathways [66]. Furthermore, sequential activation of TLR4 and TLR9 exhibits functional crosstalk that amplifies inflammatory responses [67,68]. Therefore, the LPS-induced TLR9 upregulation observed in our study likely represents a downstream regulatory event following TLR4 activation, rather than direct TLR9 ligation. Whether CTSP-W2 directly binds TLR4 and/or TLR9, or employs additional co-receptors, remains to be elucidated. Future studies using TLR-specific knockout models and competitive binding assays are warranted to precisely define the molecular targets of CTSP-W2 and to clarify its functional similarity to or divergence from LPS. Notably, molecular docking analysis revealed that both galactose and mannose chains stably bind to the TLR4/MD-2 complex (Figure 7), with internal rotational structures interacting within the groove regions of the protein, a finding consistent with previous reports [69]. This internal rotational flexibility may therefore facilitate the binding of polysaccharide molecules to PRRs. Studies have indicated that α- or β-(1→6) linked oligosaccharides with irregular branches often adopt random coil conformations owing to their considerable internal rotational freedom [70]. Aligning with this, our docking results showed that TLR4/MD2 binding to such polysaccharide motifs was highly stable, and galactoglucans with (1→6) linkages exhibited structural advantages for TLR4/MD2 receptor activation. Thus, it is reasonable to speculate that these structural characteristics may play an important role in macrophage activation [71].

## 5. Conclusions

This study presents the first systematic investigation of the immunomodulatory activity and underlying mechanisms of CTSP-W2, a homogeneous arabinogalactan glycoprotein isolated from *C. thesioides*. The glycoprotein potently enhances macrophage immune functions, including proliferation, phagocytosis, and the secretion of key inflammatory mediators (NO, TNF-α, and IL-6). Mechanistic studies revealed that CTSP-W2 promotes macrophage maturation through the TLR4/9-mediated NF-κB and MAPK signaling pathways, which was further supported by molecular docking analyses. Despite these promising findings, the present study has certain limitations. The immunomodulatory activity of CTSP-W2 was evaluated exclusively using the RAW264.7 macrophage cell line, which, while providing valuable mechanistic insights, does not fully recapitulate the complex systemic immune responses in living organisms. Consequently, in vivo studies in appropriate animal models are urgently required to assess the pharmacokinetics, biosafety, tissue distribution, and therapeutic efficacy of CTSP-W2. Building upon the current results, our future work will focus on: (1) establishing murine models (e.g., cyclophosphamide-induced immunosuppression, tumor-bearing, or infection models) to evaluate the in vivo immunomodulatory and protective effects; (2) elucidating the precise receptor-binding epitopes and the molecular basis of TLR2 versus TLR4 selectivity through surface plasmon resonance and molecular docking analyses; and (3) exploring structure-activity relationships via controlled degradation and chemical modification to optimize its immunopotency. These endeavors will pave the way for the translational development of CTSP-W2 as a safe and effective immunomodulatory agent.

## Figures and Tables

**Table 1:** Monosaccharide composition and amino acid contents of CTSP-W2.

Monosaccharide	Molar Ratio	Amino Acid	Content (µg/mg)	Amino Acid	Content (µg/mg)	Amino Acid	Content (µg/mg)
Gal	0.498	Arginine	24.45	Isoleucine	9.19	Valine	6.24
Ara	0.274	Lysine	10.41	Leucine	2.17	Serine	5.97
Man	0.086	Glutamine	35.07	Histidine	4.78	Cysteine	15.91
Glc	0.053	Asparagine	108.81	Phenylalanine	0.98	Cystine	0.62
GalA	0.052	Threonine	3.48	Glutamic acid	34.06	Proline	1.18
Xyl	0.037	Glycine	13.94	Aspartic acid	6.48	Tyrosine	2.91

**Table 2:** GC–MS analysis of the methylated derivatives of CTSP-W2.

Retention Time	Methylated Sugar	Mass Fragments (*m*/*z*)	Molar Ratio	Type of Linkage
16.401	2,3,5-Me_3_-Araf	45, 71, 87, 101, 117, 129, 145, 161	0.115	Araf-(1→
30.125	2,3-Me_2_-Araf	43, 71, 87, 99, 101, 117, 129, 161, 189	0.121	→5)-Araf-(1→
40.664	2-Me_1_-Araf	43, 58, 85, 99, 117, 127, 159, 201	0.022	→3,5)-Araf-(1→
Arabinose			0.258	
30.727	2,3,4,6-Me_4_-Galp	43, 71, 87, 101, 117, 129, 143, 161, 205	0.078	Galp-(1→
40.267	2,3,6-Me_3_-Galp	43, 87, 99, 101, 113, 117, 129, 131, 161, 173, 233	0.043	→4)-Galp-(1→
41.749	2,4,6-Me_3_-Galp	43, 87, 99, 101, 117, 129, 161, 173, 233	0.081	→3)-Galp-(1→
42.042	3,4,6-Me_3_-Galp	43, 87, 101, 129, 161, 189	0.031	→2)-Galp-(1→
46.630	2,3,4-Me_3_-Galp	43, 87, 99, 101, 117, 129, 161, 189, 233	0.118	→6)-Galp-(1→
53.146	2,3-Me_2_-Galp	43, 71, 85, 87, 99, 101, 117, 127, 159, 161, 201, 261	0.043	→4,6)-Galp-(1→
58.289	2,4-Me_2_-Galp	43, 87, 117, 129, 159, 189, 233	0.137	→3,6)-Galp-(1→
Galactose			0.531	
27.798	2,3,4,6-Me_4_-Manp	43, 71, 87, 101, 117, 129, 143, 161, 205	0.023	Manp-(1→
41.198	2,3,6-Me_3_-Manp	43, 87, 99, 101, 113, 117, 129, 131, 161, 173, 233	0.082	→4)-Manp-(1→
Mannose			0.105	
19.676	2,3,5-Me_3_-Xylp	45, 71, 87, 101, 117, 129, 145, 161	0.016	Xylp-(1→
31.478	2,3-Me_2_-Xylp	43, 71, 87, 99, 101, 117, 129, 161, 189	0.021	→4)-Xylp-(1→
Xylose			0.037	
27.472	2,3,4,6-Me_4_-Glcp	43, 71, 87, 101, 117, 129, 143, 161, 205	0.026	Glcp-(1→
38.702	2,4,6-Me_3_-Glcp	43, 87, 99, 101, 117, 129, 161, 173, 233	0.012	→3)-Glcp-(1→
Glucose			0.038	

**Table 3:** ^1^H and ^13^C NMR chemical shifts in CTSP-W2.

No.	Type of Linkage	C1/H1	C2/H2	C3/H3	C4/H4	C5/H5	C6/H6
A	α-L-Araf-(1→	107.79/5.26	81.72/4.11	76.48/3.91	81.22/3.96	61.10 3.71/3.84	
B	→5)-α-L-Araf-(1→	107.36/5.09	81.22/4.01	76.39/3.83	83.83/3.97	69.44 3.92/4.01	
C	→3,5)-α-L-Araf-(1→	107.27/5.10	82.28/3.89	81.01/4.12	82.31/4.11	69.44 3.92/4.01	
D	β-D-Galp-(1→	103.33/4.41	70.40/3.53	68.49/3.64	70.71/3.76	72.55/3.65	61.26 3.76/3.76
E	→3)-β-D-Galp-(1→	103.06/4.37	72.33/3.52	83.78/3.80	69.84/3.64	76.42/3.97	61.07 3.69/3.83
F	→4)-β-D-Galp-(1→	103.06/4.41	70.71/3.69	73.62/3.80	76.40/4.01	74.20/3.78	60.89 3.74/3.84
G	→6)-β-D-Galp-(1→	103.36/4.37	70.14/3.43	71.85/3.64	72.80/3.56	75.27/3.43	69.38 3.45/3.91
H	→3,6)-β-D-Galp-(1→	103.16/4.39	72.55/3.56	83.91/3.80	68.68/4.12	76.48/3.78	69.27 3.45/3.92
I	→4,6)-β-D-Galp-(1→	103.36/4.37	71.77/3.53	73.23/3.78	68.81/4.01	76.39 3.87	69.15 3.45/3.96
J	α-D-Xylp-(1→	96.65/4.37	72.70/3.22	73.66/3.45	75.45/3.56	60.87 3.36/3.39	
K	→4)-β-D-Xylp-(1→	103.44/4.37	71.85/3.38	72.55/3.43	75.27/3.55	62.78 3.56/3.54	
L	α-D-Manp-(1→	96.70/5.03	68.49/4.08	73.23/3.90	73.62/3.78	75.15/3.56	61.26 3.75/3.84
M	→4)-α-D-Manp-(1→	100.65/4.96	76.47/4.01	72.40/3.71	76.41/3.78	73.67/3.50	60.87 3.64/3.76
N	α-D-Glcp-(1→	92.50/5.09	72.55/3.65	75.27/3.84	69.63/3.53	76.23/3.78	61.07 3.42/3.64
O	→3)-α-D-Glcp-(1→	100.64/5.26	70.20/4.10	76.41/3.78	72.55/3.56	75.08/3.84	60.85 3.56/3.64

## Data Availability

The original contributions presented in this study are included in the article/Supplementary Materials.

## Supplementary Materials

The following supporting information can be downloaded at https://www.mdpi.com/article/10.3390/cimb48080804/s1.

## Abbreviations

AG-II	Type II arabinogalactan
AGP	Arabinogalactan protein
ANOVA	Analysis of variance
AP-1	Activator protein 1
BCA	Bicinchoninic acid
BSA	Bovine serum albumin
CCK-8	Cell Counting Kit-8
CD	Circular dichroism
COSY	Correlation spectroscopy
DEAE	Diethylaminoethyl
DMEM	Dulbecco’s modified Eagle’s medium
DMSO	Dimethyl sulfoxide
ECL	Enhanced chemiluminescence
ELISA	Enzyme-linked immunosorbent assay
ERK	Extracellular signal-regulated kinase
FBS	Fetal bovine serum
FT-IR	Fourier transform infrared
GC–MS	Gas chromatography–mass spectrometry
HMBC	Heteronuclear multiple-bond correlation
HMQC	Heteronuclear multiple-quantum correlation
HPGPC	High-performance gel permeation chromatography
HSQC	Heteronuclear single quantum coherence
IC	Ion chromatography
IκBα	Inhibitor of nuclear factor kappa B alpha
IL-1β	Interleukin-1 beta
IL-6	Interleukin-6
iNOS	Inducible nitric oxide synthase
JNKc	Jun N-terminal kinase
LPS	Lipopolysaccharide
MAPK	Mitogen-activated protein kinase
MD-2	Myeloid differentiation factor 2
MyD88	Myeloid differentiation primary response protein 88
NF-κB	Nuclear factor kappa B
NMR	Nuclear magnetic resonance
NO	Nitric oxide
NOESY	Nuclear Overhauser effect spectroscopy
PAGE	Polyacrylamide gel electrophoresis
PAS	Periodic acid–Schiff
PBS	Phosphate-buffered saline
PI	Phagocytosis index
PMSF	Phenylmethylsulfonyl fluoride
PRR	Pattern recognition receptor
PVDF	Polyvinylidene fluoride
RIPA	Radioimmunoprecipitation assay
SD	Standard deviation
SDS–PAGE	Sodium dodecyl sulfate–polyacrylamide gel electrophoresis
SEM	Scanning electron microscopy
TBST	Tris-buffered saline with Tween-20
TFA	Trifluoroacetic acid
TLR	Toll-like receptor
TNF-α	Tumor necrosis factor alpha
UV	Ultraviolet
UV-Vis	Ultraviolet–visible
