# Supplementary material for: The Characterization of a New AG-II-like Glycoprotein from Cynanchum thesioides (Freyn) K. Schum and Its Immunostimulatory Activity Through Activation of TLR4/9-Mediated MAPK/NF-κB Signaling Pathways
Source: Curr Issues Mol Biol. 2026 Aug 8;48(8):804. doi: 10.3390/cimb48080804 (PMC13510445; doi:10.3390/cimb48080804)
Supplement: Supplementary file 1 [file cimb-48-00804-s001.zip › Figure S1.pdf]

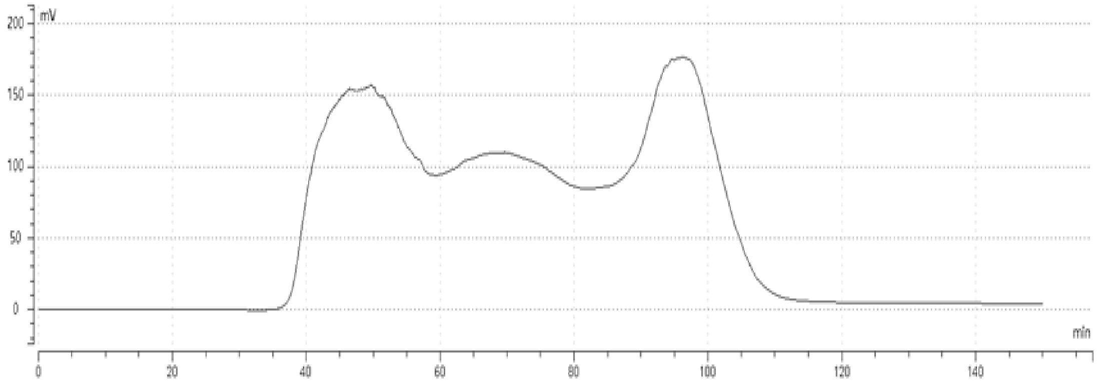

Figure S1. The gel chromatography of CTSP-W fraction by Sephadex G-100 gel column chromatography collecting three fractions with different dilution time (37-54.5min, 63-83min and 91-111min).
