# Supplementary material for: The Characterization of a New AG-II-like Glycoprotein from Cynanchum thesioides (Freyn) K. Schum and Its Immunostimulatory Activity Through Activation of TLR4/9-Mediated MAPK/NF-κB Signaling Pathways
Source: Curr Issues Mol Biol. 2026 Aug 8;48(8):804. doi: 10.3390/cimb48080804 (PMC13510445; doi:10.3390/cimb48080804)

Figure S2. The original gels of SDS–PAGE electropherogram of CTSP-W2 stained with Coomassie brilliant blue under nonreducing (a) and reducing conditions (b); lanes: M, molecular weight marker; SDS–PAGE electropherogram of CTSP-W2 stained with Coomassie brilliant blue reducing condition (c) and PAS (d); lanes: M, molecular weight marker.

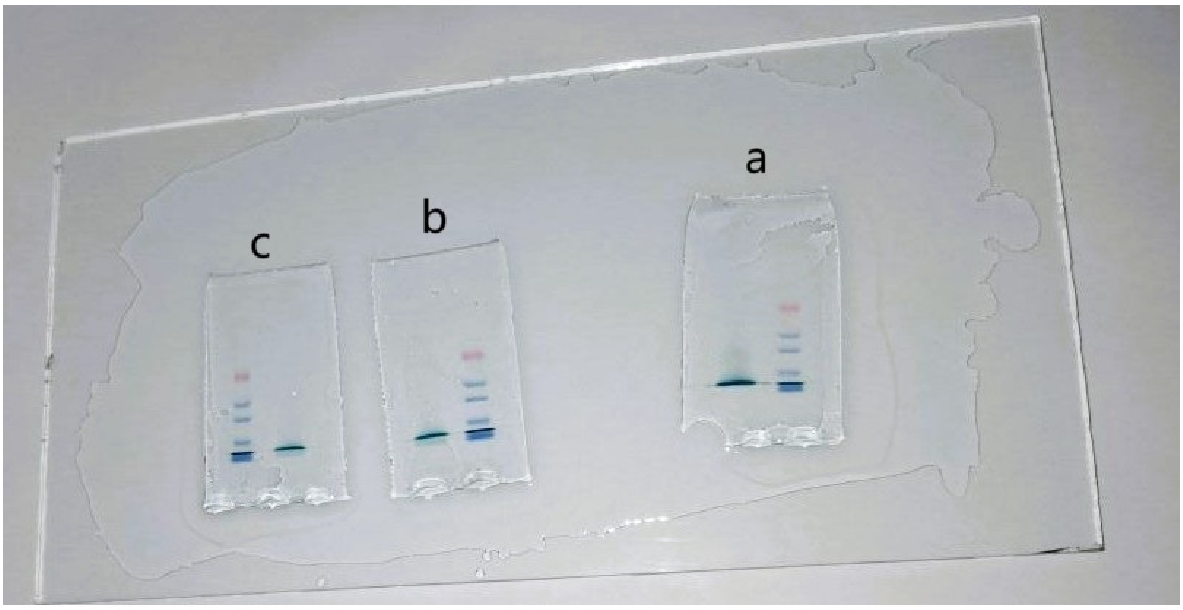

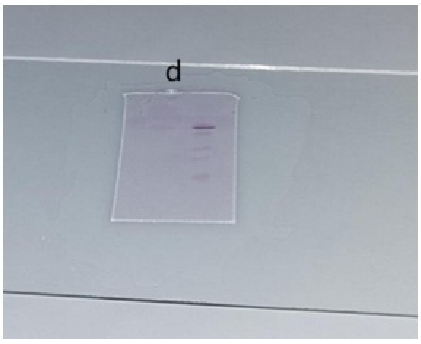

Supplement: Supplementary file 1 [file cimb-48-00804-s001.zip › Figure S2.pdf]
