# Supplementary material for: The Characterization of a New AG-II-like Glycoprotein from Cynanchum thesioides (Freyn) K. Schum and Its Immunostimulatory Activity Through Activation of TLR4/9-Mediated MAPK/NF-κB Signaling Pathways
Source: Curr Issues Mol Biol. 2026 Aug 8;48(8):804. doi: 10.3390/cimb48080804 (PMC13510445; doi:10.3390/cimb48080804)
Supplement: Supplementary file 1 [file cimb-48-00804-s001.zip › Table S1.pdf]

Docking Results and Analysis

A total of 100 docking conformations of CTSP-W2 and TLR4 were generated by the HDOCK, and the top 10 ranked models were selected for further evaluation in supplementary table 1. The predicted docking scores ranged from –624.2 to –476.1, with Model 1 achieving the most favorable docking score (–624.2) and the highest confidence score (0.9999), indicating a highly probable binding mode.

Sub. Table 1. The molecular docking results of the Top 10 ranked model

Model Rank	1	2	3	4	5	6	7	8	9	10
Docking Score	-624.2	-507.9	-498.1	-494	-493.7	-488.4	-479.1	-476.9	-476.6	-476.1
Confidence Score	0.9999	0.9992	0.9991	0.999	0.999	0.9989	0.9986	0.9986	0.9985	0.9985
Ligand rmsd (Å)	28.54	25.91	24.47	42.89	30.81	31.86	26.47	28.28	52.85	59.75
