# Supplementary material for: The Characterization of a New AG-II-like Glycoprotein from Cynanchum thesioides (Freyn) K. Schum and Its Immunostimulatory Activity Through Activation of TLR4/9-Mediated MAPK/NF-κB Signaling Pathways
Source: Curr Issues Mol Biol. 2026 Aug 8;48(8):804. doi: 10.3390/cimb48080804 (PMC13510445; doi:10.3390/cimb48080804)
Supplement: Supplementary file 1 [file cimb-48-00804-s001.zip › Table S2.pdf]

Docking Results and Analysis of TLR2

A total of 100 docking conformations of CTSP-W2 and TLR-2 were generated by the HDOCK, and the top 10 ranked models were selected for further evaluation. The predicted docking scores ranged from –483.1 to –438.6, with Model 1 achieving the most favorable docking score (–483.1) and the highest confidence score (0.9987), indicating a highly probable binding mode.

Sub. Table 2. The molecular docking results of the Top 10 ranked model

Rank	1	2	3	4	5	6	7	8	9	10
Docking Score	-483.1	-463.9	-453.7	-450.8	-446.4	-445.9	-442.4	-441.3	-441.2	-438.6
Confidence Score	0.9987	0.9981	0.9977	0.9976	0.9973	0.9973	0.9971	0.9971	0.9971	0.9969
Ligand rmsd (Å)	78.12	37.64	47.27	40.5	52.89	38.4	52.65	57.76	36.66	57.83
